# Methylation of serotonin regulating genes in cord blood cells: association with maternal metabolic parameters and correlation with methylation in peripheral blood cells during childhood and adolescence

**DOI:** 10.1186/s13148-023-01610-w

**Published:** 2024-01-03

**Authors:** Ivona Bečeheli, Marina Horvatiček, Maja Perić, Barbara Nikolić, Cyrielle Holuka, Marija Klasić, Marina Ivanišević, Mirta Starčević, Gernot Desoye, Dubravka Hranilović, Jonathan D. Turner, Jasminka Štefulj

**Affiliations:** 1 Division of Molecular Biology, Ruđer Bošković Institute, 10000 Zagreb, Croatia; 2 Department of Biology, Faculty of Science, University of Zagreb, 10000 Zagreb, Croatia; 3 Department of Infection and Immunity, Luxembourg Institute of Health, 4354 Esch-sur-Alzette, Luxembourg; 4 Faculty of Science, University of Luxembourg, 4365 Belval, Luxembourg; 5 Department of Obstetrics and Gynecology, University Hospital Centre Zagreb, 10000 Zagreb, Croatia; 6 Department of Neonatology, University Hospital Centre Zagreb, 10000 Zagreb, Croatia; 7 Department of Obstetrics and Gynecology, Medical University of Graz, 8036 Graz, Austria; 8 University Department of Psychology, Catholic University of Croatia, 10000 Zagreb, Croatia

**Keywords:** Early-life adversity, Maternal obesity, ALSPAC, Epigenetic, DoHAD, Sex differences, 5-HT

## Abstract

**Background:**

Serotonin (5-hydroxytryptamine, 5-HT) signaling is involved in neurodevelopment, mood regulation, energy metabolism, and other physiological processes. DNA methylation plays a significant role in modulating the expression of genes responsible for maintaining 5-HT balance, such as 5-HT transporter (*SLC6A4*), monoamine oxidase A (*MAOA*), and 5-HT receptor type 2A (*HTR2A*). Maternal metabolic health can influence long-term outcomes in offspring, with DNA methylation mediating these effects. We investigated associations between maternal metabolic parameters—pre-pregnancy body mass index (pBMI), gestational weight gain (GWG), and glucose tolerance status (GTS), i.e., gestational diabetes mellitus (GDM) versus normal glucose tolerance (NGT)**—**and cord blood methylation of *SLC6A4*, *MAOA*, and *HTR2A* in participants from our PlaNS birth cohort. CpG sites (15, 9, and 2 in each gene, respectively) were selected based on literature and in silico data. Methylation levels were quantified by bisulfite pyrosequencing. We also examined the stability of methylation patterns in these genes in circulating blood cells from birth to adolescence using longitudinal DNA methylation data from the ARIES database.

**Results:**

None of the 203 PlaNS mothers included in this study had preexisting diabetes, 99 were diagnosed with GDM, and 104 had NGT; all neonates were born at full term by planned Cesarean section. Methylation at most CpG sites differed between male and female newborns. *SLC6A4* methylation correlated inversely with maternal pBMI and GWG, while methylation at *HTR2A* site -1665 correlated positively with GWG. None of the maternal metabolic parameters statistically associated with *MAOA* methylation. DNA methylation data in cord blood and peripheral blood at ages 7 and 15 years were available for 808 participants from the ARIES database; 4 CpG sites (2 in *SLC6A4* and 2 in *HTR2A*) overlapped between the PlaNS and ARIES cohorts. A positive correlation between methylation levels in cord blood and peripheral blood at 7 and 15 years of age was observed for both *SLC6A4* and *HTR2A* CpG sites.

**Conclusions:**

Methylation of 5-HT regulating genes in cord blood cells is influenced by neonatal sex, with maternal metabolism playing an additional role. Inter-individual variations present in circulating blood cells at birth are still pronounced in childhood and adolescence.

**Supplementary Information:**

The online version contains supplementary material available at 10.1186/s13148-023-01610-w.

## Background

Overweight and obesity have become a serious public health burden worldwide, affecting people of all ages and socioeconomic groups [1]. One particular concern is the increase in numbers of obese and overweight women of childbearing age [2]. Women with excessive pre-pregnancy body mass index (pBMI) are at increased risk for gestational weight gain (GWG) outside the recommended range [3] and for developing gestational diabetes mellitus (GDM), a form of hyperglycemia that occurs during pregnancy [4]. Maternal overweight and obesity, inadequate GWG, and GDM have numerous negative health effects, including a long-term increased risk of obesity, diabetes and mental health problems in offspring [5–7].

Serotonin, also known as 5-hydroxytryptamine (5-HT), is a ubiquitous signaling monoamine serving as a trophic factor during development, a neurotransmitter in the brain, a gut-derived hormone, and a paracrine/autocrine messenger in various somatic tissues [8]. During development, 5-HT regulates diverse developmental processes, including the growth and maturation of its own neurons and their target regions [9]. After birth, it fine-tunes integrative brain functions and states, such as sleep–wake rhythm, appetite, cognition, mood, emotion, sexual behavior, and stress response [10]. In addition, 5-HT regulates numerous other physiological processes, including hemostasis, vascular tone, gastrointestinal functions, energy metabolism, and immune response [11].

Maintenance of 5-HT homeostasis involves multiple proteins, collectively known as 5-HT regulating proteins, which work together in a coordinated manner to support the proper balance of 5-HT activity. These proteins include membrane transporters, metabolic enzymes, and receptors for 5-HT. Solute carrier family 6 member 4 (SLC6A4), the 5-HT transporter, is the major regulator of extracellular 5-HT levels owning to its high-affinity uptake of 5-HT into cells [12]. 5-HT levels also depend on its metabolic enzymes. The rate-limiting enzyme of 5-HT catabolism is monoamine oxidase (MAO). While the two MAO isoforms, MAOA and MAOB, can act on various monoamines, MAOA has preferential affinity for 5-HT and is, therefore, the primary enzyme responsible for its degradation [13]. 5-HT receptors, including the widely expressed 5-HT receptor type 2A (HTR2A) [14], play a key role in mediating the effects of 5-HT on target cells.

Disrupted 5-HT homeostasis is a widely recognized feature of various mental health conditions [15] and an emerging component of metabolic disorders such as obesity and diabetes [16, 17]. The DOHaD (Developmental Origins of Health and Disease) concept posits DNA methylation and other epigenetic mechanisms as pivotal mediators linking prenatal adversity to lifelong risk for various chronic diseases [18, 19]. DNA methylation in genes encoding SLC6A4, MAOA and HTR2A regulates their expression [20–22] and may, thus, contribute to mediating effects of early life exposures on long-term risk for 5-HT related disorders [23]. In support of this notion, methylation of specific CpG loci within these genes in circulating blood cells collected at birth, i.e. cord blood cells, has shown predictive value for certain metabolic and mental health outcomes in childhood [24–27]. In addition, cross-sectional studies have associated DNA methylation of these genes in peripheral blood cells with a range of adverse health conditions in children, adolescents and adults [25, 28–34].

Therefore, identifying DNA methylation changes in these genes, associated with prenatal exposures, is an important first step toward understanding the mechanisms underlying developmental origins of complex disorders and developing targeted strategies to improve long-term health outcomes. We [35, 36] and others [37] have previously shown that methylation of *SLC6A4* and *HTR2A* in placenta is sensitive to maternal metabolic states in pregnancy. In cord blood cells, methylation of *SLC6A4* [25, 38, 39] and *HTR2A* [40] has been associated with GWG [25], maternal depressed mood [39], alcohol consumption [38] and exposure to green space [40] during pregnancy. However, methylation of 5-HT regulating genes in cord blood cells has not been systematically studied in the context of adverse prenatal conditions affecting pregnancy outcomes.

We hypothesize that maternal metabolic health during pregnancy may modulate methylation of 5-HT regulating genes in fetal blood cells, potentially affecting offspring health throughout their lifespan. The aim of this study was to investigate the possible predictive role of maternal metabolic parameters in determining methylation of *SLC6A4*, *MAOA*, and *HTR2A* genes in cord blood cells, using well-characterized mother-newborn pairs from our previously established Placental and Neonatal Serotonin (PlaNS) birth cohort. We specifically considered maternal anthropometric parameters (pBMI, GWG) and glucose tolerance status (GTS), i.e., GDM versus normal glucose tolerance (NGT). In addition, we used longitudinal methylation data from the Accessible Resource for Integrated Epigenomic Studies (ARIES) cohort, a subsample from the Avon Longitudinal Study of Parents and Children (ALSPAC) [41], to investigate whether inter-individual differences in methylation levels observed in circulating blood cells at birth remain stable in later life, by examining the correlation between methylation levels in cord blood and peripheral blood collected at 7 and 15 years of age.

## Results

### Characteristics of the PlaNS sample

The main characteristics of the 203 PlaNS participants involved in the study are summarized in Table 1. As per study design, participants were evenly distributed with respect to maternal GTS (NGT vs. GDM) and neonatal sex, and all neonates were born at full term by planned C-section. The values (mean ± standard deviation) of birth weight (3497 ± 481) and ponderal index (2.82 ± 0.23) were normally distributed and were similar to those reported for the Croatian population (3468 ± 458 and 2.69 ± 0.37, respectively) [42, 43]. All women with GDM (N = 99) were on an adjusted diet, 14 (14.1%) of them were treated with oral antidiabetic drugs, and 6 (6.1%) with insulin.

**Table 1:** Demographic and clinical characteristics of the PlaNS participants

Characteristic	
Maternal age at childbirth, years	34.0 [19.7–44.8]
Parity: primipara/multipara, N (%)	72 (35.5)/131 (64.5)
Pre-gestational BMI, kg/m^2^	23.4 [16.7–49.0]
Gestational weight gain, kg	13 [− 10–32]
Glucose tolerance status: NGT/GDM, N (%)	104 (51.2)/99 (48.8)
Tobacco use in pregnancy: no/yes, N (%) ^a^	135 (70.3)/57 (29.7)
Gestational age at birth, weeks	39.1 [37.0–41.6]
Newborn sex: female/male, N (%)	98 (48.3)/105 (51.7)
Newborn birth weight, g	3450 [2440–5030]
Newborn ponderal index, g/cm^3^	2.81 [2.28–3.42]

Categorical data are reported as number of subjects (N) and percentage (%). Continuous data are reported as median [range]. BMI, body mass index; GDM, gestational diabetes mellitus; NGT, normal glucose tolerance

^a^ Women who reported never having smoked or having quit smoking at least 6 months before pregnancy were categorized as non-smokers, while women who reported having smoked throughout pregnancy or having quit smoking during pregnancy were categorized as smokers; unclear cases (N = 11) were treated as missing data

The associations between maternal metabolic parameters and other characteristics of the participants are reported in Additional file 1: Tables S1A-C. Briefly, higher pBMI was associated with lower GWG (r_S_ = − 0.20, *p* = 0.004), higher birth weight (r_S_ = 0.27, *p* = 0.0001), higher ponderal index (r_S_ = 0.22, *p* = 0.002), and multiparity (*p* = 0.017). Higher GWG was associated with higher birth weight (r_S_ = 0.16, *p* = 0.024). Women with GDM had higher pBMI (*p* < 0.0001), lower GWG (*p* = 0.0001), and were slightly older (*p* = 0.023) than women with NGT. Gestational age, neonatal sex, and smoking during pregnancy were not associated with pBMI, GWG, or GTS (all *p* > 0.05).

### DNA methylation analyses in PlaNS participants

**Fig. 1:**
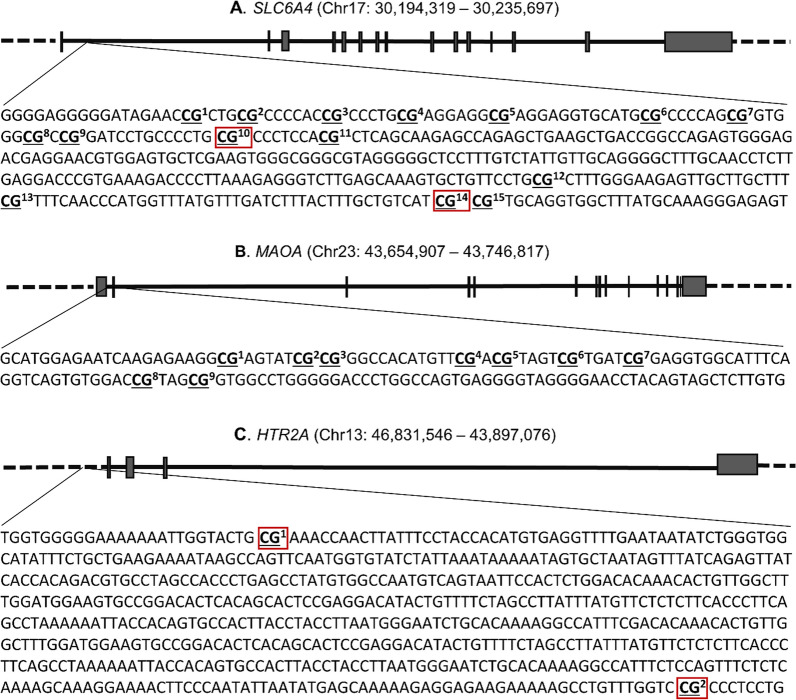
Genomic regions analyzed in the PlaNS cohort. Shown are the structures of genes encoding **A.** serotonin transporter (SLC6A4), **B.** monoamine oxidase A (MAOA), and **C.** serotonin receptor type 2A (HTR2A), along with the targeted sequences (5′–3′), starting at Chr17:30,235,549, Chr23:43,656,341, and Chr13:46,897,546, respectively (according to GRCh38.p14/hg38 assembly). Black rectangles denote exons and horizontal solid lines denote introns. The analyzed CpG sites are underlined and in bold; details of their genomic positions are provided in Additional file 1: Table S2. CpG sites covered by the Illumina Infinium Human Methylation 450K BeadChip and, therefore, available also in the ARIES database, are indicated by red rectangles

Methylation analyses in PlaNS participants targeted 15 CpG sites in *SLC6A4*, 9 CpG sites in *MAOA*, and 2 CpG sites in *HTR2A* (Fig. 1, Additional file 1: Table S2), which were selected based on literature and/or in silico findings indicating possible involvement in gene regulation and/or association with various clinical outcomes [21, 26, 44–48]. At all CpG sites except at -1665 site in *HTR2A*, methylation levels differed between female and male newborns, with higher levels in females (Additional file 1: Table S3). In a subset of participants (N = 135), who had also participated in our previous study of *HTR2A* methylation in placenta [36], we found statistically significant inverse correlation between methylation in placenta and cord blood at *HTR2A* site -1665 (r_s_ = − 0.20, *p* = 0.018). In contrast, methylation at *HTR2A* site -1224 showed no correlation between placenta and cord blood cells (r_s_ = − 0.03, *p* = 0.715).

Methylation levels of the 15 CpG sites in *SLC6A4* (Additional file 1: Figure S1A) as well as of the 9 CpG sites in *MAOA* (Additional file 1: Figure S1B) showed statistically significant positive correlations with each other, while those of the 2 CpGs in *HTR2A* were not correlated (r_s_ = 0.06, *p* = 0.370), possibly due to the larger distance between them. Therefore, in subsequent analyses, we focused on the average methylation of the 15 and 9 CpG sites in *SLC6A4* and *MAOA*, respectively, while performing separate analyses for each CpG site in *HTR2A*.

**Fig. 2:**
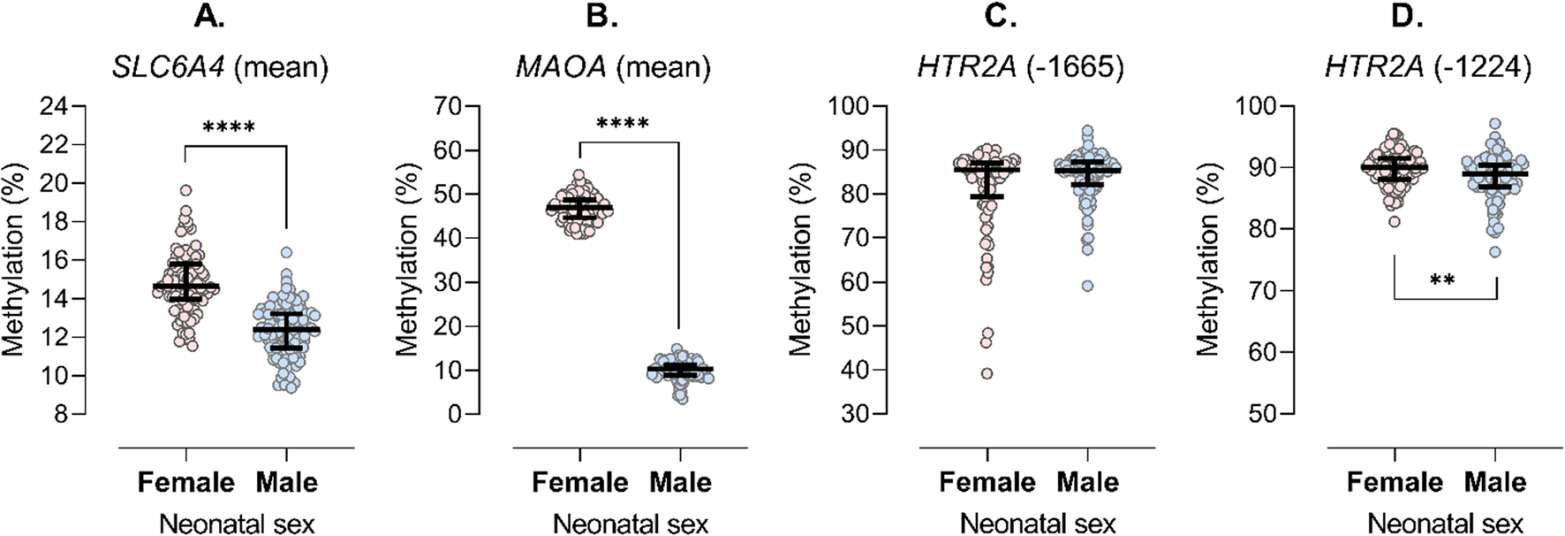
Methylation of **A.** *SLC6A4* (mean of CpG sites #1 to #15), **B.** *MAOA* (mean of CpG sites #1 to #9), and *HTR2A* sites **C.** -1665 and **D.** -1224 in cord blood cells of female and male newborns from PlaNS cohort. **p < 0.01, ****p < 0.0001

Bivariate associations of maternal and newborn characteristics with methylation levels are summarized in Additional file 1: Tables S4 and S5. Consistent with methylation levels at individual CpG sites, average *SLC6A4* methylation was about 20% higher in female than male newborns, while average *MAOA* methylation was about 4.7-fold higher in female than male newborns (Fig. 2), which could be expected given the location of *MAOA* on the X chromosome. In addition, bivariate analyses associated GWG with *SLC6A4* and *HTR2A* site -1665 methylation, parity with *SLC6A4* methylation, and birth weight with *SLC6A4*, *MAOA* and *HTR2A* site -1224 methylation, while pBMI, GTS, maternal age, tobacco use during pregnancy, gestational age, ponderal index (Additional file 1: Table S4) and cord blood cell type count (Additional file 1: Table S5) were not associated with methylation levels. In addition, in a subset of participants with available data on maternal alcohol consumption during pregnancy (N = 174), we found no association between maternal alcohol consumption and methylation levels (all *p* > 0.05).

### Association of maternal metabolic parameters with methylation

We next conducted hierarchical linear regression analysis to investigate whether maternal metabolic parameters were significant predictors of *SLC6A4*, *MAOA*, and *HTR2A* methylation in cord blood cells. Neonatal sex was included in the first step, as methylation levels differed significantly between male and female newborns. Maternal metabolic parameters (pBMI, GWG, GTS) were included in steps two to four, respectively, and control variables (parity, maternal age, birth weight), selected based on statistically significant bivariate associations with maternal metabolic parameters and/or DNA methylation, were included in the last step.

Both pBMI and GWG, but not GTS, were significant predictors of *SLC6A4* methylation, with higher pBMI and GWG associated with lower *SLC6A4* methylation (Table 2). After including control variables, pBMI and GWG remained significant predictors of *SLC6A4* methylation. On the other hand, none of the maternal metabolic parameters predicted *MAOA* methylation; only neonatal sex was significant predictor of *MAOA* methylation (Table 3). GWG was a significant predictor of methylation at *HTR2A* site -1665, although the overall model was not statistically significant (Table 4). The only significant predictor of methylation at *HTR2A* site -1224 was neonatal sex (Table 5). Replacing birth weight by ponderal index, or GTS by plasma glucose concentrations at any of the three time points of the oral glucose tolerance test, as well as performing the analyses separately in female and male newborns, did not change the results.

**Table 2:** Hierarchical linear regression analysis of maternal metabolic parameters as predictors of *SLC6A4* methylation (mean of CpG sites #1 to #15) in cord blood cells

Step	Variable	B	SE (B)	*p* (B)	R^2^	*p* (R^2^)	ΔR^2^	*p* (ΔR^2^)
1	Neonatal sex	− 2.47	0.21	< **0.0001**	0.416	< **0.0001**		
2	Neonatal sex	− 2.43	0.21	< **0.0001**	0.422	< **0.0001**	0.006	0.136
	pBMI	− 0.02	0.02	0.136				
3	Neonatal sex	− 2.39	0.21	< **0.0001**	0.441	< **0.0001**	0.019	**0.010**
	pBMI	− 0.04	0.02	**0.030**				
	GWG	− 0.05	0.02	**0.010**				
4	Neonatal sex	− 2.39	0.21	< **0.0001**	0.442	< **0.0001**	< 0.001	0.705
	pBMI	− 0.04	0.02	**0.028**				
	GWG	− 0.05	0.02	**0.014**				
	GTS	0.08	0.22	0.705				
5	Neonatal sex	− 2.38	0.21	< **0.0001**	0.459	< **0.0001**	0.018	0.100
	pBMI	− 0.04	0.02	**0.031**				
	GWG	− 0.06	0.02	**0.004**				
	GTS	0.01	0.22	0.952				
	Parity	− 0.42	0.22	0.054				
	Maternal age	0.02	0.02	0.280				
	Birth weight	0.00	0.00	0.223				

B—unstandardized beta coefficient; SE (B)—standard error of B; p (B)—significance for each of the predictors; R^2^—proportion of variance explained by predictors; p (R^2^)—significance of R^2^; ΔR^2^—increase in R^2^ resulting from the addition of predictor/s; p (ΔR^2^)—significance of ΔR^2^; pBMI—pre-pregnancy body mass index; GWG—gestational weight gain; GTS—glucose tolerance status (NGT vs. GDM, NGT as a reference). Significant *p* values are in bold

**Table 3:** Hierarchical linear regression analysis of maternal metabolic parameters as predictors of *MAOA* methylation (mean of CpG sites #1 to #9) in cord blood cells

Step	Variable	B	SE (B)	*p* (B)	R^2^	*p* (R^2^)	ΔR^2^	*p* (ΔR^2^)
1	Neonatal sex	− 29.51	1.55	< **0.0001**	0.644	< **0.0001**		
2	Neonatal sex	− 29.78	1.55	< **0.0001**	0.649	< **0.0001**	0.004	0.124
	pBMI	0.20	0.13	0.124				
3	Neonatal sex	− 29.71	1.56	< **0.0001**	0.649	< **0.0001**	0.001	0.557
	pBMI	0.17	0.13	0.189				
	GWG	− 0.08	0.14	0.557				
4	Neonatal sex	− 29.71	1.56	< **0.0001**	0.651	< **0.0001**	0.001	0.365
	pBMI	0.15	0.14	0.280				
	GWG	− 0.06	0.14	0.683				
	GTS	1.48	1.64	0.365				
5	Neonatal sex	− 30.05	1.63	< **0.0001**	0.653	< **0.0001**	0.002	0.777
	pBMI	0.11	0.14	0.440				
	GWG	− 0.06	0.15	0.675				
	GTS	1.54	1.67	0.358				
	Parity	1.48	1.66	0.375				
	Maternal age	− 0.02	0.17	0.906				
	Birth weight	0.00	0.00	0.591				

See footnotes to Table 2

**Table 4:** Hierarchical linear regression analysis of maternal metabolic parameters as predictors of cord blood methylation of CpG site #1 (-1665) in *HTR2A*

Step	Variable	B	SE (B)	*p* (B)	R^2^	*p* (R^2^)	ΔR^2^	*p* (ΔR^2^)
1	Neonatal sex	1.55	1.11	0.166	0.010	0.166		
2	Neonatal sex	1.67	1.12	0.137	0.014	0.241	0.005	0.334
	pBMI	− 0.09	0.09	0.334				
3	Neonatal sex	1.47	1.12	0.189	0.036	0.063	0.022	**0.036**
	pBMI	− 0.03	0.09	0.725				
	GWG	0.21	0.10	**0.036**				
4	Neonatal sex	1.47	1.12	0.190	0.036	0.119	< 0.001	0.791
	pBMI	− 0.03	0.10	0.776				
	GWG	0.21	0.10	**0.044**				
	GTS	− 0.31	1.17	0.791				
5	Neonatal sex	1.56	1.16	0.180	0.056	0.125	0.020	0.260
	pBMI	− 0.01	0.10	0.935				
	GWG	0.25	0.11	**0.017**				
	GTS	0.00	1.19	0.999				
	Parity	1.34	1.18	0.258				
	Maternal age	− 0.11	0.12	0.366				
	Birth weight	0.00	0.00	0.166				

See footnotes to Table 2

**Table 5:** Hierarchical linear regression analysis of maternal metabolic parameters as predictors of cord blood methylation of CpG site #2 (-1224) in *HTR2A*

Step	Variable	B	SE (B)	*p* (B)	R^2^	*p* (R^2^)	ΔR^2^	*p* (ΔR^2^)
1	Neonatal sex	− 1.32	0.44	**0.003**	0.042	**0.003**		
2	Neonatal sex	− 1.28	0.45	0.005	0.045	**0.010**	0.003	0.451
	pBMI	− 0.03	0.04	0.451				
3	Neonatal sex	− 1.27	0.45	**0.005**	0.045	**0.026**	< 0.001	0.782
	pBMI	− 0.03	0.04	0.424				
	GWG	− 0.01	0.04	0.782				
4	Neonatal sex	− 1.27	0.45	**0.005**	0.047	**0.050**	0.001	0.620
	pBMI	− 0.03	0.04	0.504				
	GWG	− 0.02	0.04	0.716				
	GTS	− 0.23	0.47	0.620				
5	Neonatal sex	− 1.21	0.47	**0.010**	0.066	0.062	0.019	0.264
	pBMI	− 0.02	0.04	0.623				
	GWG	0.00	0.04	0.946				
	GTS	− 0.16	0.48	0.734				
	Parity	0.70	0.47	0.140				
	Maternal age	− 0.01	0.05	0.868				
	Birth weight	0.00	0.00	0.175				

See footnotes to Table 2

We additionally performed exploratory analyses for each of the 15 *SLC6A4* CpG sites separately, as these sites exhibited pronounced variations in levels of methylation and different degrees of inter-individual variability. The analyses showed that GTS was a significant predictor of methylation at CpG site #1 (Additional file 1: Table S6A). Maternal anthropometric parameters (pBMI and GWG) predicted methylation at 4 CpG sites with intermediate levels of methylation and the greatest inter-individual variability: sites #12, #13, #14 and #15 (Additional file 1: Tables S6B-E). Except for site #12, pBMI statistically significantly predicted methylation only when included in the model along with GWG. Maternal metabolic characteristics were not statistically significant predictors of methylation at other CpG sites (not shown).

We sought to validate our findings using two publicly available datasets containing epigenome-wide DNA methylation data in cord blood cells along with information on neonatal sex and maternal metabolic parameters. We focused analyses on CpG sites that overlapped with those studied in the PlaNS cohort. In the GSE212174 dataset [49] containing methylation data in cord blood monocytes from a relatively small number of participants (N = 29), we replicated the association of neonatal sex with methylation of *SLC6A4* site #14 and found no association of maternal pBMI status (lean vs. obese) with methylation of *SLC6A4* sites #10 and #14 and *HTR2A* site -1664 (Additional file 1: Figure S2). However, lack of data on GWG prevented further comparison with the PlaNS results. In the GSE141065 dataset [50] containing methylation data in cord blood cells from 557 participants, we replicated the association of neonatal sex with methylation of *SLC6A4* sites #10 and #14 as well as no association of GDM with methylation of *SLC6A4* sites #10 and #14 and *HTR2A* sites -1664 and -1224 (Additional file 1: Figure S3). Data on maternal anthropometric parameters were not available for further comparison with PlaNS results.

### Association between maternal smoking and methylation

Maternal smoking is well known to affect DNA methylation in cord blood cells [51]. In a subset of women with available smoking information (N = 192), bivariate analysis did not find a significant association between maternal smoking during pregnancy and methylation (Additional file 1: Table S4). When we controlled for smoking in step 5 of hierarchical linear regression analysis, results were essentially the same for all outcomes as in the entire sample (Tables 2, 3, 4, and 5). This suggests that maternal smoking had a limited influence on methylation in our study and adds evidence to the robustness of the results with respect to the influence of neonatal sex and maternal metabolic parameters.

### Association between methylation in maternal blood and cord blood

In a subset of participants (N = 130), we examined the correlation between methylation in maternal peripheral blood cells and cord blood cells. No significant correlations were found (all p > 0.05), suggesting that methylation patterns in the examined *SLC6A4*, *MAOA*, and *HTR2A* regions are unlikely inherited from the mother, but rather influenced by prenatal environment and/or fetal genetic factors.

### Temporal stability of methylation levels

**Fig. 3:**
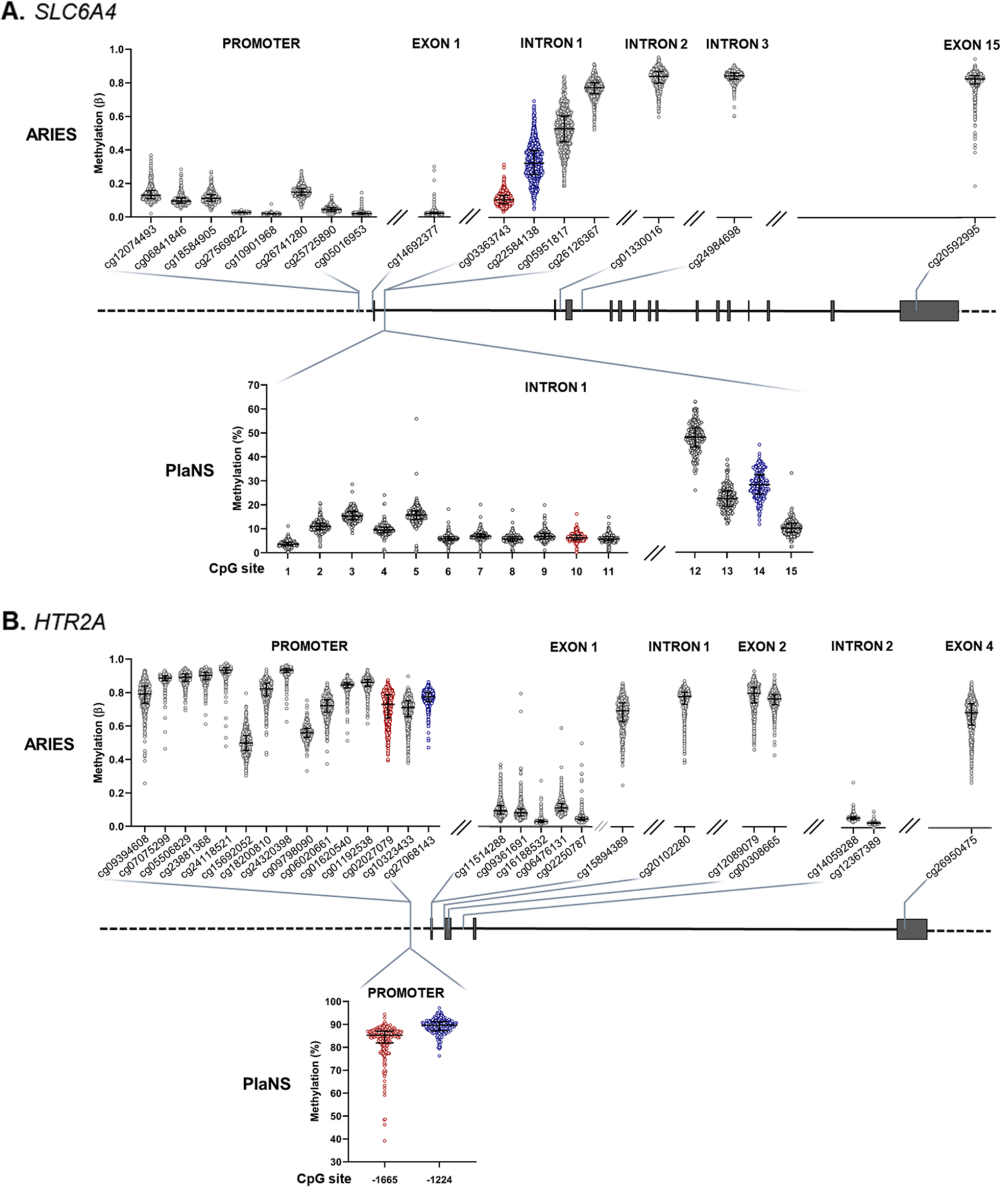
Methylation in cord blood cells, mapped to respective genomic regions in **A.** serotonin transporter (*SLC6A4*) and **B.** serotonin receptor type 2A (*HTR2A*) genes. Rectangles in gene scheme denote exons, horizontal solid lines denote introns, and dashed horizontal lines denote intergenic regions (GRCh38.p14/hg38). Methylation levels in ARIES cohort (n = 808) are shown above the gene scheme and in PlaNS cohort (n = 203) below the gene scheme, with CpG sites analyzed in both cohorts highlighted in red and blue. CpG sites in PlaNS cohort are numbered as in Fig. 1 and Additional file 1: Table S2

Next, we examined the stability of methylation levels in circulating blood cells from birth to childhood (7 years) and adolescence (15 years), using longitudinal methylation data from ARIES cohort [41]. A total of 808 participants had methylation data at all three time points and were included in the analyses. We focused on *SLC6A4* and *HTR2A* genes since their cord blood methylation showed an association with maternal metabolic traits in the PlaNS cohort. The ARIES database contains 16 CpG sites in *SLC6A4* and 27 CpG sites in *HTR2A*, 4 of which overlap with the CpG sites studied in PlaNS cohort. Consistent with PlaNS results, CpG sites within and nearby *SLC6A4* promoter region were hypomethylated compared to CpG sites within *HTR2A* promoter region (Fig. 3). Methylation between different CpG sites within each gene was either positively or negatively correlated or uncorrelated, depending on the position of the sites (Additional file 1: Figure S4).

**Fig. 4:**
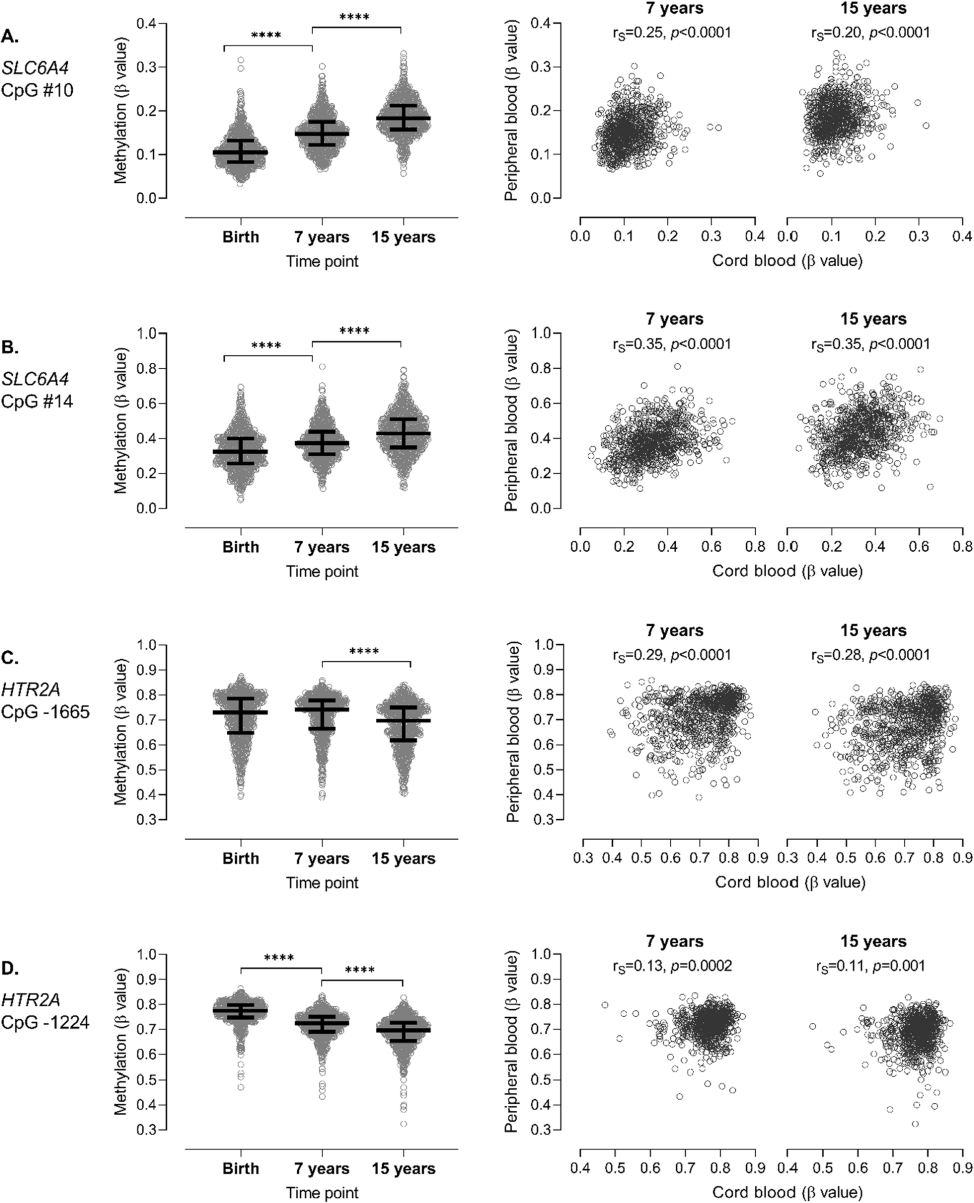
Longitudinal analyses of methylation at *SLC6A4* sites **A.** #10 (cg03363743), and **B.** #14 (cg22584138), and *HTR2A* sites **C.** -1665 (cg02027079), and **D.** -1224 (cg27068143) in ARIES cohort (N = 808). Left panels: methylation in blood cells at birth (cord blood), and at 7 and 15 years of age. ^****^p < 0.0001 (Dunn’s test following Friedman test). Right panels: methylation in peripheral blood cells at 7 and 15 years of age plotted against methylation in cord blood cells. Shown are Spearman correlation coefficient (r_s_) and statistical significance of the correlation (*p*)

Of 16 CpG sites in *SLC6A4*, only 3—including 2 that overlap with CpG sites analyzed in the PlaNS cohort, i.e. cg033637432 (#10) and cg22584138 (#14) —showed significant positive correlation of methylation between birth and ages 7 and 15 years (Table 6). Methylation of sites #10 and #14 was higher in cord blood of female than male newborns, consistent with PlaNS results, and analogous sex differences persisted during childhood and adolescence (Additional file 1: Table S7). Between time points, methylation varied on average by 32.2% at site #10 (Fig. 4A) and by 23.9% at site #14 (Fig. 4B), with methylation at both sites increasing from birth to adolescence. When analyses were performed separately for females and males, results remained essentially the same as described above for the whole sample.

**Table 6:** Correlation of *SLC6A4* methylation in circulating blood cells between birth, childhood (7 years) and adolescence (15 years) in subjects from ARIES cohort (N = 808)

Region	cg ID^a^	ID ^b^	Birth versus 7 year		Birth versus 15 year		7 year versus 15 year	
			r_s_^c^	*p* value	r_s_^c^	*p* value	r_s_^c^	*p* value
Promoter	cg12074493	/	− 0.02	0.487	− 0.02	0.653	0.05	0.135
	cg06841846	/	− 0.04	0.253	0.02	0.611	− 0.01	0.727
	cg18584905	/	0.02	0.658	− 0.01	0.698	0.05	0.188
	cg27569822	/	− 0.04	0.305	0.02	0.628	− 0.01	0.749
	cg10901968	/	0.03	0.444	− 0.01	0.821	0.07	**0.035**
	cg26741280	/	0.00	0.939	0.02	0.544	0.07	0.054
	cg25725890	/	0.09	**0.009**	0.04	0.237	0.04	0.212
	cg05016953	/	0.03	0.470	0.12	**0.001**	0.10	**0.005**
Exon 1	cg14692377	/	0.04	0.246	0.09	**0.009**	0.10	**0.005**
Intron 1	cg03363743	10	0.25	** < 0.0001**	0.20	** < 0.0001**	0.21	** < 0.0001**
	cg22584138	14	0.35	** < 0.0001**	0.35	** < 0.0001**	0.37	** < 0.0001**
	cg05951817	/	0.33	** < 0.0001**	0.24	** < 0.0001**	0.27	** < 0.0001**
	cg26126367	/	0.07	0.052	0.02	0.479	0.09	**0.014**
Intron 2	cg01330016	/	− 0.02	0.665	0.04	0.261	− 0.04	0.254
Intron 3	cg24984698	/	0.02	0.522	0.04	0.304	0.11	**0.002**
Exon 15	cg20592995	/	0.04	0.316	0.06	0.073	0.03	0.376

Significant correlations are shown in bold

^a^ Illumina ID of CpG sites

^b^ ID of CpG sites according to Fig. 1 (sites analyzed in PlaNS cohort)

^c^ Spearman’s correlation coefficient

Most of CpG sites in *HTR2A* (21 of 27)—including those studied in the PlaNS cohort (cg02027079 and cg27068143, i.e. -1665 and -1224, respectively)—showed a significant positive correlation of methylation levels between both birth and age 7 and birth and age 15 years (Table 7). Methylation varied between the three time points on average by 10.0% (site -1665) and 7.8% (site -1224), with methylation at site -1665 decreasing from age 7 to 15 years (Fig. 4C) and methylation at site -1224 already decreasing from birth to age 15 years (Fig. 4D).

**Table 7:** Correlation of *HTR2A* methylation in circulating blood cells between birth, childhood (7 years) and adolescence (15 years) in subjects from ARIES cohort (N = 808)

Region	cg ID^a^	ID#^b^	Birth versus 7 year		Birth versus 15 year		7 year versus 15 year	
			r_s_^c^	*p* value	r_s_^c^	*p* value	r_s_^c^	*p* value
Promoter	cg09394608	/	0.11	**0.001**	0.13	** < 0.0001**	0.08	**0.019**
	cg07075299	/	0.10	**0.005**	0.11	**0.002**	0.19	** < 0.0001**
	cg05506829	/	0.49	** < 0.0001**	0.47	** < 0.0001**	0.58	** < 0.0001**
	cg23881368	/	0.54	** < 0.0001**	0.51	** < 0.0001**	0.62	** < 0.0001**
	cg24118521	/	0.52	** < 0.0001**	0.45	** < 0.0001**	0.63	** < 0.0001**
	cg15692052	/	0.46	** < 0.0001**	0.40	** < 0.0001**	0.46	** < 0.0001**
	cg18200810	/	0.53	** < 0.0001**	0.46	** < 0.0001**	0.64	** < 0.0001**
	cg24320398	/	0.38	** < 0.0001**	0.37	** < 0.0001**	0.56	** < 0.0001**
	cg09798090	/	0.33	** < 0.0001**	0.32	** < 0.0001**	0.50	** < 0.0001**
	cg06020661	/	0.27	** < 0.0001**	0.23	** < 0.0001**	0.41	** < 0.0001**
	cg01620540	/	0.03	0.474	0.01	0.851	0.11	**0.003**
	cg01192538	/	0.08	**0.029**	0.05	0.149	0.09	**0.007**
	cg02027079	1	0.29	** < 0.0001**	0.28	** < 0.0001**	0.30	** < 0.0001**
	cg10323433	/	0.10	**0.004**	0.11	**0.002**	0.21	** < 0.0001**
	cg27068143	2	0.13	**0.0002**	0.11	**0.001**	0.28	** < 0.0001**
Exon 1	cg11514288	/	0.16	** < 0.0001**	0.18	** < 0.0001**	0.26	** < 0.0001**
	cg09361691	/	0.21	** < 0.0001**	0.19	** < 0.0001**	0.23	** < 0.0001**
	cg16188532	/	0.19	** < 0.0001**	0.14	** < 0.0001**	0.19	** < 0.0001**
	cg06476131	/	0.23	** < 0.0001**	0.12	**0.001**	0.20	** < 0.0001**
	cg02250787	/	0.15	** < 0.0001**	0.14	** < 0.0001**	0.24	** < 0.0001**
	cg15894389	/	0.24	** < 0.0001**	0.21	** < 0.0001**	0.38	** < 0.0001**
Intron 1	cg20102280	/	0.08	**0.022**	0.04	0.306	0.25	** < 0.0001**
Exon 2	cg12089079	/	0.11	**0.002**	0.09	**0.007**	0.15	** < 0.0001**
	cg00308665	/	0.16	** < 0.0001**	0.10	**0.004**	0.28	** < 0.0001**
Intron 2	cg14059288	/	0.04	0.253	0.05	0.156	− 0.02	0.648
	cg12367389	/	0.06	0.066	0.09	**0.013**	0.09	**0.015**
Exon 4	cg26950475	/	0.08	**0.025**	0.07	0.057	0.04	0.206

Significant correlations are shown in bold

^a^Illumina ID of CpG sites

^b^ID of CpG sites according to Fig. 1 (sites analyzed in PlaNS cohort)

^c^Spearman’s correlation coefficient

### Association between genetic variants and methylation

We used the methylation quantitative trait loci (mQTLs) database [52] to examine in ARIES cohort a possible association between genetic variants and inter-individual variations in methylation of *SLC6A4* and *HTR2A* genes. In cord blood cells, mQTLs were identified for 2 of 16 available CpGs in *SLC6A4* and for 13 of 27 available CpGs in *HTR2A* (Additional file 1: Figure S5). However, no mQTLs were identified for CpGs that overlapped with those we had studied in the PlaNS cohort, i.e. sites #10 and #14 (cg03363743 and cg22584138, respectively) in *SLC6A4* and sites -1665 and -1224 (cg02027079 and cg27068143, respectively) in *HTR2A*. We extended the search to later time points (7 and 15 years of age). At age 7 years, mQTLs were identified for 2 CpGs in *SLC6A4* and 15 CpGs in *HTR2A*, while at age 15 years, mQTLs were identified for 2 CpGs in *SLC6A4* and 20 CpGs in *HTR2A*. At all three time points (birth, age 7 and 15 years), the average number of mQTLs per CpG site was higher for *HTR2A* than *SLC6A4*. Taken together, the data indicate a lack of association between genetic variants and inter-individual variation in cord blood methylation of 4 CpG sites studied in the PlaNS cohort. They also suggest a generally stronger genetic influence on *HTR2A* than on *SLC6A4* methylation.

### Association between *SLC6A4* methylation and expression in cord blood cells

To examine the potential regulatory role of cord blood methylation of our genes of interest, we examined the correlation of methylation and expression levels in cord blood cells using publicly available datasets from ENVIR*ON*AGE cohort participants (Gene Expression Omnibus accession numbers: GSE151042 and GSE151373) [53]. Data on *MAOA* methylation and *HTR2A* expression were not available, so we restricted the analysis to *SLC6A4*. The relative expression levels of *SLC6A4*, calculated by dividing the normalized signal intensities of *SLC6A4* by that of beta-actin (*ACTB*), showed a positive correlation with methylation at cg22584138 (CpG #14), one of the sites associated with maternal metabolic parameters in the PlaNS cohort (r = 0.18, *p* = 0.030, N = 150). No significant correlation was found with other sites examined (Additional file 1: Table S8).

## Discussion

Our results in mother-infant pairs from the PlaNS cohort show that maternal metabolic health, in addition to a substantial contribution of neonatal sex, is a modest but significant predictor of *SLC6A4* and *HTR2A* methylation but not *MAOA* methylation in cord blood cells. The results remained significant after including several control variables in the model. In an independent cohort (ARIES), inter-individual differences in *HTR2A* methylation in circulating blood cells remained relatively stable from birth throughout childhood and adolescence, while stability for *SLC6A4* was limited to some CpG sites.

Previous studies have linked *SLC6A4* methylation in cord blood cells to maternal factors such as parity and gestational weight gain [25], as well as alcohol consumption [38] and depressed mood [39] during pregnancy. We found that maternal anthropometric parameters both before and during pregnancy were weakly but significantly associated with *SLC6A4* methylation in cord blood cells. Specifically, higher pBMI and higher GWG predicted lower *SLC6A4* methylation. These results are partially consistent with a previous study that found a negative correlation between cord blood *SLC6A4* methylation and GWG but not pBMI [25]. In our study, pBMI emerged as a significant predictor of *SLC6A4* methylation only when it was included in the regression model together with GWG. This finding suggests a combined association of pBMI and GWG with *SLC6A4* methylation and highlights the importance of maternal metabolic health both before and during pregnancy.

In addition, we found that GDM diagnosis predicted higher methylation at a specific CpG site in *SLC6A4* (site #1). Interestingly, this site is the closest to the *SLC6A4* promoter region at which methylation in the placenta showed a positive correlation with maternal blood glucose concentration during pregnancy [19]. Taken together, these results suggest a possible differential sensitivity of specific CpG sites in *SLC6A4* to various metabolic disturbances.

Methylation of *HTR2A* site -1665 correlated positively with GWG. Although this correlation remained statistically significant even after inclusion of various control variables, it should be noted that none of the linear regression models with -1665 methylation as an outcome reached statistical significance. On the other hand, *MAOA* methylation in cord blood cells was not associated with any of the maternal metabolic characteristics. It is worth noting that while *SLC6A4* and *HTR2A* are highly specific for regulating and maintaining 5-HT signaling, *MAOA* catabolizes not only 5-HT but also other biogenic monoamines.

The effect size of maternal metabolic parameters on *SLC6A4* and *HTR2A* methylation was rather modest. For example, for each one-unit increase in pBMI (kg/m^2^) and GWG (kg), there was a corresponding increase of 0.11 and 0.15 methylation units (%), respectively, at CpG site #14 in *SLC6A4* gene. Modest effect sizes are common in studies examining the association of prenatal exposures and DNA methylation. However, despite their small magnitudes, these effects are both statistically significant and highly reproducible [54]. Notably, even subtle changes in DNA methylation may associate with differences in neonatal phenotype [47].

Diagnosis of GDM was not a significant predictor of *HTR2A* methylation in cord blood cells. This result was somewhat unexpected considering previous findings on the association of GDM with methylation of *HTR2A* [36, 37] in placental tissue. In addition to the tissue-specific effects of the metabolic environment on DNA methylation processes, this discrepancy could be accounted for by the direct exposure of placental trophoblast cells to maternal circulation [19], different from circulating fetal blood cells, which are directly exposed only to fetal influences that may change as a consequence of maternal exposures.

In agreement with a previous report [45], our study confirmed higher *SLC6A4* methylation in cord blood cells of female compared to male newborns in both PlaNS and ARIES cohorts. Sex-dependent differences in *SLC6A4* methylation persisted throughout childhood and adolescence in ARIES participants. Thus, CpG sites in *SLC6A4* are among the 5% of autosomal CpGs with stable sex differences during the first two decades of life [55].

Differences between males and females in *SLC6A4* methylation in peripheral blood cells [56–58] as well as in density/function of SLC6A4 in the brain [59] have been observed also in adults. These findings are consistent with numerous studies reporting sex-specific differences in clinical presentation [60–62] and prevalence [61, 63] of 5-HT related disorders. Given that sex-dependent changes in *SLC6A4* methylation occur early in development and that 5-HT has a profound regulatory role in neurodevelopment [9], one could speculate that *SLC6A4* methylation, through modulation of *SLC6A4* expression [44], may influence sex-specific neurodevelopmental outcomes and potentially play a role in sex-dependent disparities in 5-HT related disorders.

Methylation of *MAOA*, an X-linked gene, was remarkably higher in female than male newborns, likely due to DNA methylation-mediated silencing of an *MAOA* allele on the inactive X chromosome [64]. This is consistent with reports in adult peripheral blood cells [65, 66]. We also observed a small but significant sex-dependent difference in methylation of *HTR2A* site -1224 in cord blood samples from PlaNS participants, which is consistent with our previous findings in placenta [36].

A previous study reported a lack of correlation of *SLC6A4* methylation (mean of 27 CpG sites) in peripheral blood cells between 5 and 10 years of age, suggesting that inter-individual differences in *SLC6A4* methylation are not maintained during childhood [67]. Our results in ARIES cohort are partially consistent with this finding as for most CpG sites in *SLC6A4* we did not find correlation between different time points. However, 3 CpG sites—including site #14 (cg22584138), which was associated with maternal anthropometric parameters and correlated with *SLC6A4* expression levels in cord blood cells—showed a significant positive correlation of methylation between birth and childhood (7 years) and birth and adolescence (15 years). The same 3 and additional 5 CpG sites showed a positive correlation between childhood and adolescence. A possible reason for the partial discrepancy could be the different tissue used, i.e., buccal cells [67] versus circulating blood cells (ARIES data).

Most of *HTR2A* CpG sites examined (21 of 27), including sites -1665 and -1224 studied in PlaNS cohort, showed significant positive correlation of methylation levels between birth and ages 7 and 15 years, while 25 of 27 sites showed significant positive correlation between ages 7 and 15 years. This suggests substantial stability of inter-individual differences in *HTR2A* methylation in circulating blood cells from birth up to adolescence.

Interestingly, methylation of *HTR2A* promoter sites decreased over time, either continuously from birth to adolescence (-1224) or only from childhood to adolescence (-1665), while methylation of *SLC6A4* intron 1 sites increased continuously from birth to adolescence. Recent epigenome-wide DNA methylation results suggest that CpG sites with decreasing DNA methylation during the first two decades of life are enriched in pathways related to immune system development, while CpG sites with increasing methylation during the first two decades of life are enriched in pathways related to neurodevelopment [55]. Taken together, the observed opposing changes in *SLC6A4* and *HTR2A* methylation could potentially indicate distinct roles of these genes during maturation.

Our study has several notable strengths. First, we investigated a well-characterized mother-newborn pairs from the PlaNS cohort. We ensured equal representation of women with NGT and GDM and considered maternal anthropometric characteristics both before and during pregnancy. All neonates were born under uniform conditions by planned cesarean section at full term (at week ≥ 37.0), eliminating potential confounding factors associated with variations in birth mode or gestational age, and had no known fetal or neonatal anomalies. We included various control variables in our statistical models and conducted a sensitivity analysis using a subset of participants, which allowed us to assess the consistency and robustness of our models.

We must also acknowledge limitations of our study. First, we did not measure certain factors that have been associated with methylation of specific loci in cord blood cells and, therefore, could potentially influence our results, such as maternal exposure to environmental endocrine disruptors [68] and green space [40] during pregnancy or maternal adverse childhood experiences [69]. We excluded women with clinical depression, but attention should be also paid to maternal depressed mood during pregnancy [39].

Second, our approach based on CpG candidates may have omitted CpG sites whose methylation level could be related to the maternal traits of interest. Nevertheless, the selection of CpG loci was based on their demonstrated functional relevance [21, 26, 44–48] and in silico analyses for putative transcription factor binding sites. Focusing on specific CpG loci also minimizes the possibility of type I errors that can arise from testing a large number of functionally non-significant CpG loci, a recognized challenge in epigenome-wide association studies [70].

Third, correction of DNA methylation data for cellular heterogeneity was performed only in the ARIES cohort. However, in a subset of PlaNS participants (N = 122) with available blood cell count data, we found no significant associations of cord blood cell counts with DNA methylation or maternal metabolic variables of interest. This finding reduces the likelihood of cellular heterogeneity having confounded our results.

Fourth, we used an independent cohort (ARIES) to examine the persistence of inter-individual differences in DNA methylation from birth to adolescence. Future follow-up studies with PlaNS participants will verify the stability of methylation of 5-HT regulating genes over time, while also investigating whether methylation variations at birth can serve as predictive biomarkers for later developmental outcomes. Finally, we attempted to replicate the analyses using publicly available cord blood DNA methylation datasets [49, 50]. However, they lacked certain variables that were essential for our study, such as gestational weight gain. Although our study requires replication, it still contributes valuable new data to the growing understanding of 5-HT regulation during early human development. We hope it will stimulate and guide future research in this area.

## Conclusions

Neonatal sex was a stronger predictor of methylation of 5-HT regulating genes in cord blood cells than maternal metabolic traits considered in the study. This highlights that sex-dependent differences in 5-HT regulating genes occur early during human development. Maternal anthropometric parameters before and/or during pregnancy were modest but significant predictors of *SLC6A4* and *HTR2A* but not *MAOA* methylation in cord blood cells. Our results also indicate that inter-individual differences in methylation of specific CpG sites in *SLC6A4* and *HTR2A*, which could be attributed to the prenatal metabolic environment, are stable during postnatal development until adolescence. It remains to be determined whether they contribute to the mechanisms by which suboptimal maternal metabolic health translates into offspring susceptibility to 5-HT-related disorders.

## Methods

### PlaNS participants

The study included a subset of participants from the PlaNS (Placental and Neonatal Serotonin) birth cohort study, which is ongoing in Zagreb, Croatia (project code: IP-2018-01-6547; December 1, 2018). Purpose and design of the PlaNS cohort study have been described previously [36]. Briefly, pregnant women were recruited at the Department of Gynecology and Obstetrics, University Hospital Centre Zagreb, Zagreb, Croatia, 1–3 days before planned C-section. Participants’ demographic and clinical data were obtained from medical records and questionnaires completed by mothers. Maternal metabolic variables of interest included pBMI, GWG and GTS (NGT vs. GDM). Additional variables considered in the present study (Table 1) were selected on the basis of their previously demonstrated associations with DNA methylation in cord blood cells [25, 54, 71]. pBMI was calculated by dividing pre-pregnancy body weight in kilograms by height in meters squared. GWG was obtained from medical records and verified by using the self-reported data in the questionnaires. GDM diagnosis was based on the criteria proposed by the International Association of Diabetes and Pregnancy Study Groups (IADPSG) guidelines [72] implemented in Croatian clinical setting [73]. Gestational age was determined as the number of weeks from the first day of the mother's last menstrual period and, if needed, is adjusted according to established guidelines [74]. Ponderal index (PI) was calculated using formula: birthweight (g) × 100/(birth length (cm))^3^.

The present study was designed to include approximately equal numbers of women with normal glucose tolerance (NGT) and with GDM, as well as approximately equal numbers of female and male newborns. Inclusion criteria were singleton pregnancy, absence of any pre-existing diabetes prior to pregnancy, maternal age between 18 and 45 years, pBMI ≥ 16.0 kg/m2, gestational age at birth ≥ 37.0 weeks, absence of diagnosed or suspected intrauterine growth restriction, and no known congenital anomalies. None of the women involved in the study suffered from depression or took antidepressant before or during pregnancy, factors previously associated with methylation of specific CpG loci in cord blood cells [75]. The original sample included 206 mother-newborn pairs. Methylation data from 3 participants did not meet pyrosequencing quality control or were identified as outliers using the ROUT method [76]. These participants were excluded from all subsequent analyses, resulting in a final sample of 203 mother-newborn dyads.

The study was approved by the Ethics Committee of University Clinical Hospital Centre Zagreb (class: 8.1–18/162–2, number: 02/21 AG; approved on 18.07.2018) and the Bioethics Committee of the Ruđer Bošković Institute, Zagreb (BEP-8761/2–2018; approved on 26.11.2018). All participants gave written informed consent to participate in the study. All procedures were in accordance with the Declaration of Helsinki.

### Sample collection, DNA isolation, and DNA methylation analyses

Cord blood samples (≈ 4 mL) were collected in a K_3_EDTA VACUETTE® Blood Collection Tube (Greiner Bio-One, Kremsmuenster, Austria) by puncture of the umbilical vein after birth, and were immediately mixed by inverting the tube. Whenever possible, sample aliquots were analyzed for hemogram using the DxH 500 Hematology Analyzer (Beckman Coulter, Brea, CA, USA). Aliquots of cord blood used for isolation of genomic DNA (2 mL) were stored in cryotubes at − 80 °C for up to 6 months prior to DNA isolation. Genomic DNA was extracted from whole blood samples using the DNA Isolation Kit for Mammalian Blood (Roche, Basel, Switzerland) according to the manufacturer’s protocol. Integrity of isolated DNA was assessed by 0.8% agarose gel electrophoresis, while yield and purity were determined by spectrophotometry (NanoPhotometer® N60/N50, Implen, Germany). DNA samples were stored in aliquots at − 80 °C until further processing.

DNA methylation of targeted CpG sites in 5-HT regulating genes (Additional file 1: Table S2) was quantified by bisulfite pyrosequencing. Sequences of primers used in PCR and pyrosequencing (Additional file 1: Table S9) were designed using PyroMark Assay Design Software (version 2.0, Qiagen, Hilden, Germany) or taken from literature [33, 47, 48, 77]. All steps were performed according to the manufacturer's protocol and recommendations. In brief, uniform amounts of isolated genomic DNA (800 ng per sample) were bisulfite converted using the EZ DNA Methylation-Gold Kit (Zymo Research, Irvine, CA, USA). Converted DNA was amplified using the PyroMark PCR Kit (Qiagen, Hilden, Germany). PCR products were checked for specificity by 2% agarose gel electrophoresis and then pyrosequenced using PyroMark Q24 Advanced CpG Reagents on the PyroMark Q24 Advanced Pyrosequencing System (both from Qiagen, Hilden, Germany). All assays included a negative control and a reference sample. Pyrosequencing quality control was performed for all samples using PyroMark Q24 Advanced Software (version 3.0.0, Qiagen, Hilden, Germany). When methylation levels were statistically significantly correlated between CpG sites, we performed statistical analyses using their average value as an integrative measure of DNA methylation in the region of interest.

### ARIES cohort analysis

Changes in methylation of 5-HT regulating genes in circulating blood cells from birth to childhood and adolescence were investigated using data from publicly available Accessible Resource for Integrated Epigenomic Studies (ARIES) database, which provides longitudinal epigenome-wide methylation data from 1018 mother–child pairs [41]. In children, DNA methylation was measured in whole blood samples collected at birth (cord blood) and at age 7 and 15 years (peripheral blood). The inclusion criterion for our study was the availability of DNA methylation data at all three time points, resulting in a sample size of 808 participants.

DNA methylation in ARIES participants was quantified by the Illumina Infinium HumanMethylation 450K BeadChip Assay (Illumina 450K Array; from Illumina Inc., San Diego, CA, USA). This assay covers over 450,000 CpG sites across the human genome, including 16 CpG sites in *SLC6A4* and 27 CpG sites in *HTR2A*, which were of interest in our study. Procedures for blood sample collection and storage, DNA extraction, DNA methylation analysis, and details of data pre-processing, normalization, and correction for batch effects and cellular heterogeneity have been described elsewhere [78, 79]. Statistical analyses were performed using beta (β) value, the ratio between the intensity of the methylated probe and the total intensity, where 0 represents an unmethylated cytosine and 1 represents a fully methylated cytosine.

ARIES is a sub-study of the Avon Longitudinal Study of Parents and Children (ALSPAC) [78]. Ethical approval for the study was obtained from the ALSPAC Ethics and Law Committee and the Local Research Ethics Committees. Consent for biological samples has been collected in accordance with the Human Tissue Act (2004). Informed consent for the use of data collected via questionnaires and clinics was obtained from participants following the recommendations of the ALSPAC Ethics and Law Committee at the time.

### Statistical analyses

Normality of the data distribution was tested using the D’Agostino-Pearson normality test. Potential outliers were screened for using the ROUT method [76]. Continuous variables with normal distribution were compared using Student’s t test, with Welch’s correction where appropriate. Continuous variables with non-normal distribution were compared using Mann–Whitney test. Bivariate correlations were analyzed with the Pearson correlation coefficient (r_p_) in the case of normally distributed data or the Spearman correlation coefficient (r_s_) in the case of non-normally distributed data. Fisher’s exact test (FET) was used to test for differences in frequency distributions.

Hierarchical linear regression analysis was used to examine the relationship between maternal metabolic parameters as predictors and methylation of 5-HT regulating genes in cord blood cells as a continuous outcome, controlling for selected maternal and newborns’ characteristics. We chose a hierarchical framework with sequential input of maternal metabolic parameters (pBMI, GWG, GTS) to assess their incremental contribution to methylation variance and test, based on ΔR^2^, whether successive models fit better than previous ones. The suitability of the data for parametric analyses and collinearity between predictors were tested before the main analysis. Methylation data (percentages) that deviated from a normal distribution were rank-transformed to achieve normality for use in regression analysis. The statistical power was assessed using G*Power software (version 3.1, Heinrich Heine University, Duesseldorf, Germany) [80]. With a sample size of 203, 7 predictors, a medium effect size, and an α-probability error of 5%, the statistical power was 90.5%.

Statistical analyses were performed using IBM SPSS Statistics 21.0 for Windows (SPSS Statistics, Chicago, IL, USA), GraphPad Prism 8 (GraphPad Software Inc, San Diego, CA, USA) and R, R V.3.4.4 (The R Foundation for Statistical Computing) using the “rstatix” and “corrplot” packages. All statistical tests were two-sided with a threshold of significance set at *p* ≤ 0.05.

### Supplementary Information

**Additional file 1: Tables S1A–C**. Associations between maternal metabolic parameters and other sample characteristics in PlaNS cohort. **Table S1A**. Association between maternal anthropometric parameters and continuous sample characteristics. **Table S1B**. Association between maternal anthropometric parameters and categorical sample characteristics. **Table S1C**. Association between maternal glucose tolerance status and other sample characteristics. **Table S2**. CpG sites targeted by methylation analyses in PlaNS cohort. **Table S3**. Methylation levels (%) at individual CpG sites in PlaNS participants. **Table S4**. Significance levels (*p* values) for bivariate associations of maternal and neonatal characteristics with methylation of serotonin regulating genes in cord blood cells of PlaNS participants.** Table S5**. Significance levels (*p* values) for correlation between cord blood cell type count and methylation of serotonin regulating genes in cord blood cells in a subset of PlaNS participants (N = 122). **Tables S6A-S6E**. Hierarchical linear regression analysis of maternal metabolic parameters as predictors of methylation at individual CpG sites in *SLC6A4* gene in cord blood cells of PlaNS participants. **Table S6A**. Maternal metabolic parameters as predictors of methylation at CpG site #1 in *SLC6A4* gene. **Table S6B**. Maternal metabolic parameters as predictors of methylation at CpG site #12 in *SLC6A4* gene.**Table S6C**. Maternal metabolic parameters as predictors of methylation at CpG site #13 in *SLC6A4* gene.**Table S6D**. Maternal metabolic parameters as predictors of methylation at CpG site #14 in *SLC6A4* gene. **Table S6E**. Maternal metabolic parameters as predictors of methylation at CpG site #15 in *SLC6A4* gene.**Table S7**. Methylation levels (β values) in ARIES participants at CpG sites overlapping with CpG sites analyzed in PlaNS cohort. **Table S8**. Correlation between *SLC6A4* methylation and expression in cord blood cells. **Table S9**. Primers used in DNA methylation analysis by bisulfite pyrosequencing in PlaNS cohort. **Figure S1.** Correlation between methylation levels at CpG sites in (A) *SLC6A4* and (B) MAOA, as observed in the PlaNS cohort. **Figure S2**. Association of newborn sex and maternal pre-pregnancy body weight status with methylation of* SLC6A4* and *HTR2A* CpG sites in cord blood monocytes, as determined in GSE212174 dataset [49]. **Figure S3**. Association of newborn sex and maternal glucose tolerance status with methylation of *SLC6A4* and *HTR2A* CpG sites in cord blood cells, as determined in GSE141065 dataset [50]. **Figure S4**. Correlation between methylation levels at CpG sites in **A** *SLC6A4* and **B** *HTR2A*, as observed in the ARIES cohort. **Figure S5**. Temporal mapping of methylation quantitative trait loci (mQTLs) for Illumina 450K CpG sites in **A** *SLC6A4* and **B** *HTR2A* genes in the ARIES cohort.

## Abbreviations

5-HT: 5-Hydroxytryptamine (serotonin)
ACTB: Beta actin
ALSPAC: Avon Longitudinal Study of Parents and Children
ARIES: Accessible Resource for Integrated Epigenomic Studies
BMI: Body mass index
DOHaD: Developmental Origins of Health and Disease
FET: Fisher’s exact test
GDM: Gestational diabetes mellitus
GTS: Glucose tolerance status
GWG: Gestational weight gain
HTR2A: 5-HT receptor type 2A
IADPSG: International Association of Diabetes and Pregnancy Study Groups
MAO: Monoamine oxidase
MAOA: Monoamine oxidase A
MAOB: Monoamine oxidase B
mQTLs: Methylation quantitative trait loci
NGT: Normal glucose tolerance
pBMI: Pre-pregnancy body mass index
PI: Ponderal index
PlaNS: Placental and neonatal serotonin
SLC6A4: 5-HT transporter
