# Supplementary material for: Methylation of serotonin regulating genes in cord blood cells: association with maternal metabolic parameters and correlation with methylation in peripheral blood cells during childhood and adolescence
Source: Clin Epigenetics. 2024 Jan 3;16:4. doi: 10.1186/s13148-023-01610-w (PMC10765867; doi:10.1186/s13148-023-01610-w)
Supplement: Supplementary file 1 — Additional file 1: Tables S1A–C. Associations between maternal metabolic parameters and other sample characteristics in PlaNS cohort. Table S1A. Association between maternal anthropometric parameters and continuous sample characteristics. Table S1B. Association between maternal anthropometric parameters and categorical sample characteristics. Table S1C. Association between maternal glucose tolerance status and other sample characteristics. Table S2. CpG sites targeted by methylation analyses in PlaNS cohort. Table S3. Methylation levels (%) at individual CpG sites in PlaNS participants. Table S4. Significance levels (p values) for bivariate associations of maternal and neonatal characteristics with methylation of serotonin regulating genes in cord blood cells of PlaNS participants. Table S5. Significance levels (p values) for correlation between cord blood cell type count and methylation of serotonin regulating genes in cord blood cells in a subset of PlaNS participants (N = 122). Tables S6A-S6E. Hierarchical linear regression analysis of maternal metabolic parameters as predictors of methylation at individual CpG sites in SLC6A4 gene in cord blood cells of PlaNS participants. Table S6A. Maternal metabolic parameters as predictors of methylation at CpG site #1 in SLC6A4 gene. Table S6B. Maternal metabolic parameters as predictors of methylation at CpG site #12 in SLC6A4 gene.Table S6C. Maternal metabolic parameters as predictors of methylation at CpG site #13 in SLC6A4 gene.Table S6D. Maternal metabolic parameters as predictors of methylation at CpG site #14 in SLC6A4 gene. Table S6E. Maternal metabolic parameters as predictors of methylation at CpG site #15 in SLC6A4 gene.Table S7. Methylation levels (β values) in ARIES participants at CpG sites overlapping with CpG sites analyzed in PlaNS cohort. Table S8. Correlation between SLC6A4 methylation and expression in cord blood cells. Table S9. Primers used in DNA methylation analysis by bisulfite pyrosequencing in Pla [file 13148_2023_1610_MOESM1_ESM.pdf]

Supplementary Tables

Tables S1A-S1C. Associations between maternal metabolic parameters and other sample characteristics in PlaNS cohort.

Table S1A. Association between maternal anthropometric parameters and continuous sample characteristics.

	pBMI		GWG	
	r_s	*p*-value	r_s	*p*-value
GWG	-0.20	**0.004**		
Birth weight	0.27	**<0.0001**	0.16	**0.024**
Ponderal index	0.22	**0.002**	0.10	0.173
Maternal age	0.04	0.578	0.003	0.963
Gestational age	-0.05	0.506	0.08	0.286

Statistically significant findings are shown in bold. GWG, gestational weight gain; pBMI, pre-gestational body mass index; r_S, Spearman's correlation coefficient.

Table S1B. Association between maternal anthropometric parameters and categorical sample characteristics.

	N	pBMI (kg/m^2)	GWG (kg)
**Parity**			
primipara	72	22.6 [20.9−26.2]	14 [11−17]
multipara	131	24.6 [21.6−29.4]	13 [10−16]
p-value		**0.017**	0.162
**Glucose tolerance status**			
normal glucose tolerance	104	22.6 [20.8−26.0]	14 [11−18]
gestational diabetes mellitus	99	25.9 [22.2−31.4]	12 [8−15]
p-value		**<0.0001**	**0.0001**
**Newborn sex**			
female	98	23.2 [21.3−26.9]	13 [10−16]
male	105	24.1 [21.6−29.1]	13 [10−17]
p-value		0.255	0.550
**Smoking in pregnancy**			
no	135	23.1 [21.3−29.1]	13 [10−16]
yes	57	23.4 [21.6−27.6]	14 [11−17]
p-value		0.993	0.100

Data are reported as median [interquartile range]. *p*-values for the difference between the groups were determined using Mann-Whitney test. Statistically significant differences are in bold. GWG, gestational weight gain; pBMI, pre-gestational body mass index.

Table S1C. Association between maternal glucose tolerance status and other sample characteristics.

	NGT (N = 104)	GDM (N = 99)	*p*-value
Maternal BMI before pregnancy, kg/m^2	22.6 [20.8-26.0]	25.9 [22.2-31.4]	**<0.0001** [b]
Weight gain during pregnancy, kg	14.3 [11.0-18.0]	12.0 [8.0-15.0]	**0.0001** [b]
Maternal age at childbirth, years	33.2 [29.7-36.6]	34.2 [31.4-38.0]	**0.023** [c]
Gestational age at childbirth, weeks	39.3 [38.8-39.7]	39.1 [38.6-39.6]	0.141 [c]
Newborn birth weight, g	3425 [3193-3810]	3500 [3180-3820]	0.322 [c]
Newborn ponderal index, g/cm^3	2.81 [2.70-2.97]	2.81 [2.66-3.02]	0.393 [d]
Parity (primipara/multipara), N (%)	38 (36.5)/66 (63.5)	34 (34.3)/65(65.7)	0.771 [d]
Newborn sex (female/male), N (%)	51 (49.0)/53 (51.0)	47 (47.5)/52 (52.5)	0.889 [d]
Tobacco use in pregnancy (no/yes), N (%) [a]	71 (71.7)/28 (28.3)	64 (68.8)/29 (31.2)	0.752 [d]

NGT, normal glucose tolerance; GDM, gestational diabetes mellitus; BMI, body mass index.
Continuous data are reported as median [interquartile range]. Categorical data are reported as number of subjects (N) and percentage (%).
[a] Women who reported never having smoked or having quit smoking at least 6 months before pregnancy were categorized as non-smokers, while women who reported having smoked throughout pregnancy or having quit smoking during pregnancy were categorized as smokers; unclear cases (5 NGT, 6 GDM) were treated as missing data.
p-values for the difference between the NGT and GDM groups were determined using [b]Mann-Whitney test, [c]Student's t-test, or [d]Fisher's exact test. Statistically significant differences are in bold.

Table S2. CpG sites targeted by methylation analyses in PlaNS cohort.

Gene	CpG site #	Chr	GRCh38 coordinates [a]	RefSeq coordinates [b]	Location in gene	TSS (bp) postition [c]	Illumina ID [d]
SLC6A4	1	17	Chr17: 30 235 532	5405	Intron 1	-13573	/
	2	17	Chr17: 30 235 527	5410	Intron 1	-13568	/
	3	17	Chr17: 30 235 519	5418	Intron 1	-13560	/
	4	17	Chr17: 30 235 512	5425	Intron 1	-13553	/
	5	17	Chr17: 30 235 504	5433	Intron 1	-13545	/
	6	17	Chr17: 30 235 490	5447	Intron 1	-13531	/
	7	17	Chr17: 30 235 482	5455	Intron 1	-13523	/
	8	17	Chr17: 30 235 475	5462	Intron 1	-13516	/
	9	17	Chr17: 30 235 472	5465	Intron 1	-13513	/
	10	17	Chr17: 30 235 457	5480	Intron 1	-13498	cg03363743
	11	17	Chr17: 30 235 448	5489	Intron 1	-13489	/
	12	17	Chr17: 30 235 272	5665	Intron 1	-13313	/
	13	17	Chr17: 30 235 247	5690	Intron 1	-13288	/
	14	17	Chr17: 30 235 203	5734	Intron 1	-13244	cg22584138
	15	17	Chr17: 30 235 201	5736	Intron 1	-13242	/
MAOA	1	X	Chr23: 43 656 361	5201	Exon 1	18	/
	2	X	Chr23: 43 656 368	5208	Exon 1	25	/
	3	X	Chr23: 43 656 370	5210	Exon 1	27	/
	4	X	Chr23: 43 656 383	5223	Exon 1	40	/
	5	X	Chr23: 43 656 386	5226	Exon 1	43	/
	6	X	Chr23: 43 656 392	5232	Exon 1	49	/
	7	X	Chr23: 43 656 398	5238	Exon 1	55	/
	8	X	Chr23: 43 656 427	5267	Intron 1	84	/
	9	X	Chr23: 43 656 432	5272	Intron 1	89	/
HTR2A	1	13	Chr13: 46 897 571	4464	Promoter	-1665	cg02027079
	2	13	Chr13: 46 897 130	4905	Promoter	-1224	cg27068143

Chr, chromosome.
[a] Position in Genome Reference Consortium human genome build GRCh38.p14/hg38
[b] Position in gene-specific reference sequence (RefSeq) NG_011747.2, NG_008957.2, and NG_013011.1 for *SLC6A4*, *MAOA*, and *HTR2A*, respectively.
[c] Position in base pairs (bp) relative to translation start site (TSS).
[d] CpG locus designation in the Illumina CG database.

Table S3. Methylation levels (%) at individual CpG sites in PlaNS participants.

Gene	CpG site #	Integral sample (N=203)	Females (N=98)	Males (N=105)	*p*-value
SLC6A4	1	3.54 [2.91-4.34]	3.87 [3.10-4.71]	3.25 [2.56-3.86]	**<0.0001**[a]
	2	10.99 [9.65-12.32]	11.70 [10.48-13.20]	10.14 [8.87-11.47]	**<0.0001**[a]
	3	15.28 [13.74-17.25]	15.99 [14.58-18.06]	14.51 [13.55-16.16]	**<0.0001**[a]
	4	9.53 [8.41-10.68]	10.16 [9.24-11.20]	9.00 [7.97-10.01]	**<0.0001**[a]
	5	15.72 [13.89-17.50]	16.88 [15.41-18.24]	14.59 [12.99-16.02]	**<0.0001**[a]
	6	5.81 [5.03-6.68]	6.35 [5.76-7.23]	5.40 [4.65-6.00]	**<0.0001**[a]
	7	6.90 [6.15-7.82]	7.45 [6.66-8.47]	6.53 [5.94-7.20]	**<0.0001**[a]
	8	5.73 [4.93-6.70]	6.35 [5.47-7.32]	5.37 [4.59-6.03]	**<0.0001**[a]
	9	6.79 [5.79-8.08]	7.45 [6.69-8.67]	6.30 [5.58-7.09]	**<0.0001**[a]
	10	6.22 [5.41-7.36]	6.99 [6.16-7.92]	5.71 [5.01-6.32]	**<0.0001**[a]
	11	5.76 [5.00-6.68]	6.42 [5.64-7.27]	5.18 [4.62-5.94]	**<0.0001**[a]
	12	47.96 [43.89-51.86]	51.83 [48.75-54.61]	45.03 [41.22-47.49]	**<0.0001**[a]
	13	22.40 [19.12-25.61]	25.61 [22.79-28.18]	19.88 [17.37-22.27]	**<0.0001**[a]
	14	28.18 [24.27-32.45]	31.62 [28.82-35.46]	25.11 [22.01-27.14]	**<0.0001**[b]
	15	10.08 [8.26-11.93]	11.85 [10.15-13.40]	8.47 [7.38-10.14]	**<0.0001**[a]
MAOA	1	24.02 [16.44-44.62]	44.71 [41.08-48.85]	16.90 [14.38-19.93]	**<0.0001**[a]
	2	15.03 [8.50-55.56]	55.67 [52.78-57.67]	8.67 [6.96-10.55]	**<0.0001**[a]
	3	13.47 [7.68-32.34]	32.44 [27.80-34.41]	7.71 [6.06-9.48]	**<0.0001**[a]
	4	9.84 [6.07-46.77]	46.99 [41.99-51.03]	6.19 [4.72-7.66]	**<0.0001**[b]
	5	19.53 [10.47-54.88]	54.90 [52.32-57.55]	10.52 [8.98-12.45]	**<0.0001**[a]
	6	13.30 [6.47-46.00]	46.09 [42.03-49.88]	6.55 [4.81-7.95]	**<0.0001**[a]
	7	18.25 [12.41-60.73]	60.84[58.69-63.01]	12.46 [10.26-14.49]	**<0.0001**[a]
	8	17.50 [11.38-58.23]	58.36 [56.65-61.05]	11.42 [9.73-12.90]	**<0.0001**[a]
	9	13.30 [9.20-21.74]	21.74 [19.06-24.57]	9.27 [7.76-11.01]	**<0.0001**[a]
HTR2A	1	85.35 [81.91-87.07]	85.46 [79.39-86.98]	85.28 [82.13-87.26]	0.334[a]
	2	89.67 [87.30-91.23]	90.02 [88.05-91.52]	88.90 [86.82-90.41]	**0.004**[a]

Data are reported as median [interquartile range]. N, number of subjects. *p*-values for the difference between female and male newborns were determined using [a]Mann-Whitney test or [b]Student's t-test. Statistically significant differences are in bold.

Table S4. Significance levels (*p*-values) for bivariate associations of maternal and neonatal characteristics with methylation of serotonin regulating genes in cord blood cells of PlaNS participants.

	SLC6A4 (average)	***MAOA*** (average)	***HTR2A*** (-1665)	***HTR2A*** (-1224)
**pBMI**	0.161	0.720	0.979	0.263
**GWG**	**0.038**	0.385	**0.022**	0.213
**GTS**	0.869	0.737	0.407	0.543
**PAR**	**0.017**	0.628	0.329	0.420
**MAD**	0.053	0.146	0.269	0.947
**TOB**	0.482	0.618	0.053	0.623
**SEX**	**<0.0001**	**<0.0001**	0.334	**0.004**
**GA**	0.244	0.244	0.315	0.081
**BW**	**0.043**	**0.008**	0.558	**0.019**
**PI**	0.743	0.714	0.361	0.294

Shown are *p*-values for the association of maternal pre-pregnancy body mass index (pBMI), gestational weight gain (GWG), glucose tolerance status (GTS; normoglycemia vs. gestational diabetes mellitus), parity (PAR; primiparous vs. multiparous), maternal age at childbirth (MAD), maternal smoking during pregnancy (TOB), neonatal sex (SEX), gestational age (GA), birth weight (BW) and ponderal index (PI) with methylation of *SLC6A4* (average of CpG sites #1 to #15), *MAOA* (average of CpG sites #1 to 9), and *HTR2A* (sites -1665 and -1224) in cord blood cells. Number of participants was 203, except for TOB, where it was 192 due to missing values. *p*-values were determined by Student's t test, Mann-Whitney test, Pearson correlation, or Spearman correlation, as appropriate. Statistically significant associations are in bold. In analyses separated by neonatal sex, no significant associations were found between *MAOA* methylation and any of maternal or neonatal characteristics.

Table S5. Significance levels (*p*-values) for correlations between cord blood cell type count and methylation of serotonin regulating genes in cord blood cells in a subset of PlaNS participants (N=122).

	SLC6A4 (average)	***MAOA*** (average)	***HTR2A*** (-1665)	***HTR2A*** (-1224)
**Lymphocytes**	0.178	0.654	0.538	0.062
**Monocytes**	0.223	0.525	0.923	0.619
**Neutrophils**	0.166	0.043	0.706	0.416
**Eosinophils**	0.893	0.748	0.414	0.197
**Basophils**	0.365	0.707	0.371	0.150

Shown are *p*-values for correlation of lymphocyte, monocyte, neutrophil, eosinophil and basophil counts in cord blood with methylation of *SLC6A4* (average of CpG sites #1 to #15), *MAOA* (average of CpG sites #1 to #9), and *HTR2A* (sites -1665 and -1224) in cord blood cells of subjects with available hematogram data (N=122). *p*-values were determined by Pearson or Spearman correlation, as appropriate. In analyses separated by neonatal sex, no significant associations were found between cell type counts and methylation levels.

Tables S6A-S6E. Hierarchical linear regression analysis of maternal metabolic parameters as predictors of methylation at individual CpG sites in *SLC6A4* gene in cord blood cells of PlaNS participants.

Table S6A. Maternal metabolic parameters as predictors of methylation at CpG site #1 in *SLC6A4* gene.

Step	Variable	B	SE (B)	*p* (B)	R^2	*p* (R^2)	ΔR^2	*p* (ΔR^2)
1	Neonatal sex	-0.86	0.18	<**0.0001**	0.099	<**0.0001**		
2	Neonatal sex	-0.85	0.18	<**0.0001**	0.099	<**0.0001**	<0.0001	0.856
	pBMI	0.00	0.02	0.856				
3	Neonatal sex	-0.83	0.18	<**0.0001**	0.109	<**0.0001**	0.010	0.144
	pBMI	-0.01	0.02	0.561				
	GWG	-0.02	0.02	0.144				
4	Neonatal sex	-0.83	0.18	<**0.0001**	0.162	<**0.0001**	0.053	<**0.0001**
	pBMI	-0.02	0.02	0.173				
	GWG	-0.01	0.02	0.412				
	GTS	0.67	0.19	<**0.0001**				
5	Neonatal sex	-0.86	0.18	<**0.0001**	0.189	<**0.0001**	0.027	0.096
	pBMI	-0.03	0.02	0.115				
	GWG	-0.02	0.02	0.175				
	GTS	0.61	0.19	**0.001**				
	Parity	-0.29	0.19	0.126				
	Maternal age	0.01	0.02	0.447				
	Birth weight	0.00	0.00	0.062				

B, unstandardized beta coefficient; SE (B), standard error of B; *p* (B), significance for each of the predictors; R^2, proportion of variance explained by predictors; *p* (R^2), significance of $R^{2;}$ ΔR^2, increase in R^2 resulting from the addition of predictor/s; *p* (ΔR^2), significance of ΔR^2; pBMI, pre-pregnancy body mass index; GWG, gestational weight gain; GTS, glucose tolerance status (normal glucose tolerance (NGT) vs. gestational diabetes mellitus, with NGT as a reference). Significant *p* values are in bold.

Table S6B. Maternal metabolic parameters as predictors of methylation at CpG site #12 in *SLC6A4* gene.

Step	Variable	B	SE (B)	*p* (B)	R^2	*p* (R^2)	ΔR^2	*p* (ΔR^2)
1	Neonatal sex	-7.68	0.66	<**0.0001**	0.403	<**0.0001**		
2	Neonatal sex	-7.49	0.66	<**0.0001**	0.422	<**0.0001**	0.020	**0.010**
	pBMI	-0.14	0.05	**0.010**				
3	Neonatal sex	-7.35	0.65	<**0.0001**	0.440	<**0.0001**	0.017	**0.014**
	pBMI	-0.18	0.06	**0.002**				
	GWG	-0.15	0.06	**0.014**				
4	Neonatal sex	-7.35	0.65	**<0.0001**	0.440	<**0.0001**	0.001	0.647
	pBMI	-0.18	0.06	**0.001**				
	GWG	-0.14	0.06	**0.020**				
	GTS	0.31	0.68	0.647				
5	Neonatal sex	-7.31	0.68	<**0.0001**	0.442	<**0.0001**	0.002	0.872
	pBMI	-0.18	0.06	**0.003**				
	GWG	-0.15	0.06	**0.017**				
	GTS	0.25	0.70	0.724				
	Parity	-0.52	0.69	0.459				
	Maternal age	0.00	0.00	0.789				
	Birth weight	0.02	0.07	0.753				

See footnotes to Table S6A.

Table S6C. Maternal metabolic parameters as predictors of methylation at CpG site #13 in *SLC6A4* gene.

Step	Variable	B	SE (B)	*p* (B)	R^2	*p* (R^2)	ΔR^2	*p* (ΔR^2)
1	Neonatal sex	-5.67	0.55	**<0.0001**	0.350	<**0.0001**		
2	Neonatal sex	-5.53	0.55	**<0.0001**	0.363	<**0.0001**	0.012	0.051
	pBMI	-0.09	0.05	0.051				
3	Neonatal sex	-5.40	0.54	**<0.0001**	0.386	<**0.0001**	0.023	**0.007**
	pBMI	-0.12	0.05	**0.008**				
	GWG	-0.13	0.05	**0.007**				
4	Neonatal sex	-5.40	0.54	**<0.0001**	0.387	<**0.0001**	0.001	0.624
	pBMI	-0.12	0.05	**0.013**				
	GWG	-0.14	0.05	**0.006**				
	GTS	-0.28	0.57	0.624				
5	Neonatal sex	-5.25	0.55	**<0.0001**	0.409	<**0.0001**	0.022	0.064
	pBMI	-0.11	0.05	**0.030**				
	GWG	-0.16	0.05	**0.002**				
	GTS	-0.43	0.57	0.448				
	Parity	-1.43	0.57	**0.012**				
	Maternal age	0.06	0.06	0.319				
	Birth weight	0.00	0.00	0.676				

See footnotes to Table S6A.

Table S6B. Maternal metabolic parameters as predictors of methylation at CpG site #14 in *SLC6A4* gene.

Step	Variable	B	SE (B)	*p* (B)	R^2	*p* (R^2)	ΔR^2	*p* (ΔR^2)
1	Neonatal sex	-7.26	0.63	<**0.0001**	0.401	<**0.0001**		
2	Neonatal sex	-7.17	0.63	<**0.0001**	0.405	<**0.0001**	0.005	0.208
	pBMI	-0.07	0.05	0.208				
3	Neonatal sex	-7.02	0.62	<**0.0001**	0.427	<**0.0001**	0.021	**0.007**
	pBMI	-0.10	0.05	**0.049**				
	GWG	-0.15	0.06	**0.007**				
4	Neonatal sex	-7.02	0.62	<**0.0001**	0.427	<**0.0001**	<0.0001	0.789
	pBMI	-0.11	0.05	**0.048**				
	GWG	-0.15	0.06	**0.010**				
	GTS	0.18	0.65	0.789				
5	Neonatal sex	-7.03	0.64	<**0.0001**	0.441	<**0.0001**	0.014	0.183
	pBMI	-0.12	0.06	**0.037**				
	GWG	-0.17	0.06	**0.003**				
	GTS	-0.05	0.66	0.946				
	Parity	-0.71	0.66	0.279				
	Maternal age	0.00	0.00	0.178				
	Birth weight	0.09	0.07	0.174				

See footnotes to Table S6A.

Table S6E. Maternal metabolic parameters as predictors of methylation at CpG site #15 in *SLC6A4* gene.

Step	Variable	B	SE (B)	*p* (B)	R^2	*p* (R^2)	ΔR^2	*p* (ΔR^2)
1	Neonatal sex	-3.63	0.36	**<0.0001**	0.336	<**0.0001**		
2	Neonatal sex	-3.58	0.36	**<0.0001**	0.341	<**0.0001**	0.005	0.228
	pBMI	-0.04	0.03	0.228				
3	Neonatal sex	-3.48	0.36	**<0.0001**	0.373	<**0.0001**	0.033	**0.002**
	pBMI	-0.06	0.03	**0.038**				
	GWG	-0.10	0.03	**0.002**				
4	Neonatal sex	-3.48	0.35	**<0.0001**	0.381	<**0.0001**	0.007	0.126
	pBMI	-0.07	0.03	**0.019**				
	GWG	-0.09	0.03	**0.005**				
	GTS	0.57	0.37	0.126				
5	Neonatal sex	-3.46	0.37	**<0.0001**	0.385	<**0.0001**	0.004	0.753
	pBMI	-0.07	0.03	**0.023**				
	GWG	-0.10	0.03	**0.003**				
	GTS	0.50	0.38	0.185				
	Parity	-0.23	0.38	0.541				
	Maternal age	0.03	0.04	0.434				
	Birth weight	0.00	0.00	0.642				

See footnotes to Table S6A.

Table S7. Methylation levels (β values) in ARIES participants at CpG sites overlapping with CpG sites analyzed in PlaNS cohort.

Gene	CpG site [a]	Time point	Integral sample	Females	Males	*p*-value
SLC6A4	#10	Birth	0.11 [0.08-0.13]	0.12 [0.09-0.14]	0.09 [0.08-0.12]	**<0.0001** [c]
		7 years	0.15 [0.12-0.17]	0.15 [0.13-0.19]	0.15 [0.12-0.17]	**<0.0001** [c]
		15 years	0.18 [0.16-0.21]	0.20 [0.17-0.22]	0.17 [0.15-0.20]	**<0.0001** [d]
		p-value [b]	**<0.0001**	**<0.0001**	**<0.0001**	
	#14	Birth	0.33 [0.26-0.40]	0.37 [0.31-0.44]	0.28 [0.22-0.35]	**<0.0001** [c]
		7 years	0.37 [0.31-0.44]	0.41 [0.36-0.48]	0.34 [0.28-0.40]	**<0.0001** [c]
		15 years	0.43 [0.35-0.51]	0.48 [0.41-0.55]	0.38 [0.32-0.46]	**<0.0001** [c]
		p-value [b]	**<0.0001**	**<0.0001**	**<0.0001**	
HTR2A	#1	Birth	0.73 [0.65-0.79]	0.73 [0.65-0.78]	0.74 [0.65-0.79]	0.375 [c]
		7 years	0.74 [0.66-0.78]	0.75 [0.67-0.78]	0.74 [0.67-0.77]	0.125 [c]
		15 years	0.70 [0.62-0.75]	0.70 [0.62-0.74]	0.70 [0.61-0.75]	0.722 [c]
		p-value [b]	**<0.0001**	**<0.0001**	**<0.0001**	
	#2	Birth	0.77 [0.75-0.80]	0.77 [0.75-0.80]	0.77 [0.75-0.80]	0.975 [c]
		7 years	0.72 [0.69-0.75]	0.72 [0.69-0.75]	0.73 [0.69-0.75]	0.109 [c]
		15 years	0.70 [0.65-0.73]	0.69 [0.65-0.72]	0.70 [0.65-0.73]	0.340 [c]
		p-value [b]	**<0.0001**	**<0.0001**	**<0.0001**	

Data are reported as median [interquartile range]. [a] CpG sites #10, #14, #1, and #2 correspond to Illumina IDs cg03363743, cg22584138, cg02027079, and cg27068143, respectively. [b] *p*-values for the difference among different time points were determined using Friedman test. *p*-values for the difference between female and male newborns were determined using [c] Mann-Whitney test or [d] Student's t-test. Statistically significant differences are in bold.

Table S8. Correlation between *SLC6A4* methylation and expression in cord blood cells. Data were obtained from publicly available datasets of participants in the ENVIR*ON*AGE cohort (N=150) [1]. Expression levels were expressed as normalized signal intensities of *SLC6A4* relative to normalized signal intensities of *ACTB*.

**Region**	**cg ID** [a]	**ID#** [b]	**r_s** [c]	***p*-value**
Promoter	cg12074493	/	0.09	0.306
	cg06841846	/	0.11	0.182
	cg18584905	/	0.04	0.667
	cg27569822	/	-0.01	0.931
	cg10901968	/	-0.03	0.757
	cg26741280	/	0.00	0.998
	cg25725890	/	0.03	0.688
	cg05016953	/	-0.03	0.684
Exon 1	cg14692377	/	-0.03	0.706
Intron 1	cg03363743	10	0.04	0.595
	cg22584138	14	**0.18**	**0.030**
	cg05951817	/	0.02	0.770
	cg26126367	/	0.02	0.787
Intron 2	cg01330016	/	0.01	0.910
Intron 3	cg24984698	/	0.12	0.131
Exon 15	cg20592995	/	0.04	0.668

[a] Illumina identifier (ID) of CpG sites;
[b] ID of CpG sites according to **Table S2** (sites analyzed in PlaNS cohort);
[c] Spearman's correlation coefficient.
Significant correlations are shown in bold.

Table S9. Primers used in DNA methylation analyses by bisulfite pyrosequencing in PlaNS cohort.

Gene	CpG site #	Primer sequence (5' - 3')	GRCh38.p14/hg38 coordinates	Product size (bp)	Sequence source
SLC6A4	1–11	forward: GGGGAGGGGGATAGAAT	Chr17: 30 235 549 - 30 235 533	273	PyroMark Assay Design Software, version 2.0
		reverse (biotinylated): ACAACACTTTACTCAAAACCCTCTTTA	Chr17: 30 235 302 - 30 235 276		
		sequencing: GGGGAGGGGGATAGAAT	Chr17: 30 235 549 - 30 235 533		
	12–15	forward: TTTTAAAGAGGGTTTTGAGTAAAGTG	Chr17: 30 235 306 - 30 235 281	150	PyroMark Assay Design Software, version 2.0
		reverse-biotinylated: TTTATATCAACCAAAACTCTCCCTTTACAT	Chr17: 30 235 185 - 30 235 156		
		sequencing: GGGTTTTGAGTAAAGTGT	Chr17: 30 235 297 - 30 235 280		
MAOA	1–9	forward: GGGGAGTTGATAGAAGGGTTTTTTTTAT	Chr23: 43 656 261 - 43 656 288	353	[2,3]
		reverse (biotinylated): TATATCTACCTCCCCCAATCACACC	Chr23: 43 656 590 - 43 656 614		
		sequencing: AGTTAAAGTATGGAGAATTAAG	Chr23: 43 656 333 - 43 656 354		
HTR2A	1	forward: TGGTGGGGGAAAAAAATT	Chr13: 46 897 896 - 46 897 879	383	[4]
		reverse (biotinylated): AAATAACCTTTTATACAAATTCCC	Chr13: 46 897 236 - 46 897 213		
		sequencing: TGGTGGGGGAAAAAAATT	Chr13: 46 897 896 - 46 897 879		
	2	forward: GATATAAATATTGTTGGTTTTGGATGGA	Chr13: 46 897 377 - 46 897 850	276	[5]
		reverse (biotinylated): ACTACAAAATAACAACAACCAAAAA	Chr13: 46 897 125 - 46 897 101		
		sequencing: GGGAGAAGAAAAAGTTTG	Chr13: 46 897 155 - 46 897 138		

bp, base pair

Supplementary Figures

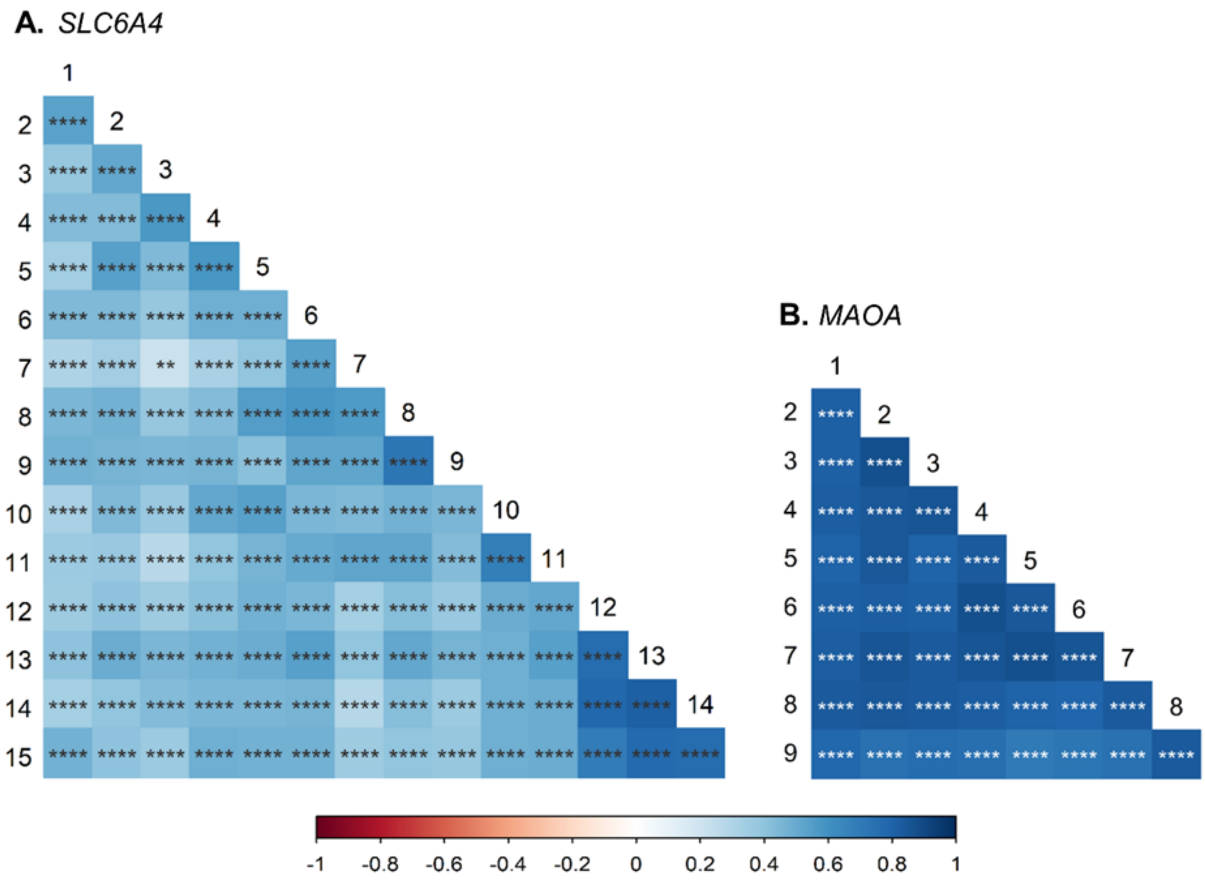

Figure S1. Correlation between methylation levels at CpG sites in **A.** *SLC6A4* and **B.** *MAOA*, as observed in the PlaNS cohort. The row and column numbers denote CpG sites as listed in **Table S2**. The color of each square denotes the strength of correlation calculated by Spearman correlation coefficient, as indicated in the legend. Asterisks within each square indicate corresponding *p*-values: ***p*<0.01, *****p*<0.0001. The graph was created using the "corrplot" package of the R software [6].

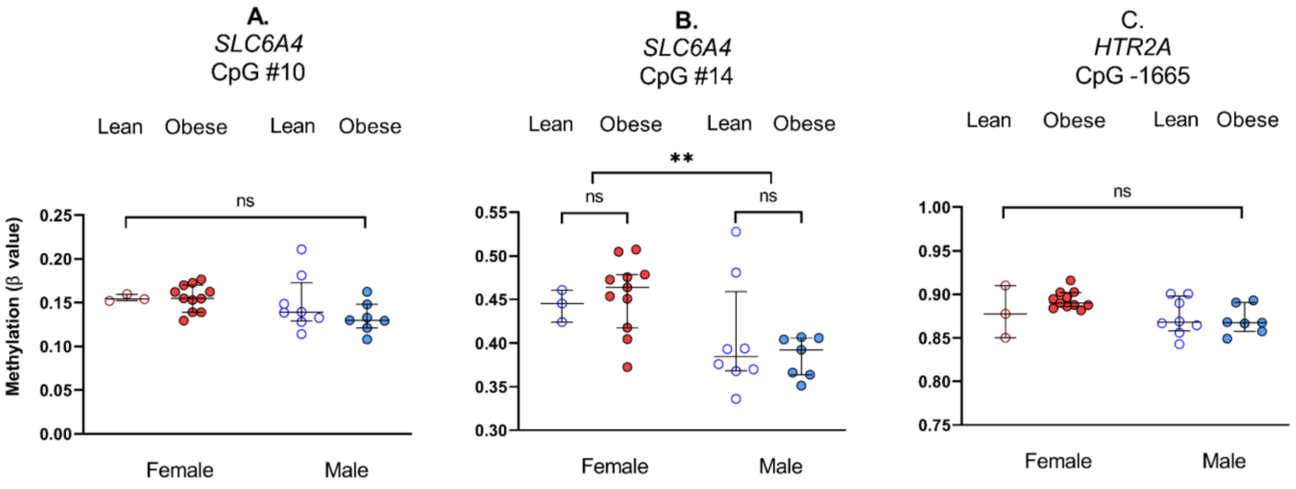

Figure S2. Association of newborn sex and maternal pre-pregnancy body weight status with methylation of *SLC6A4* and *HTR2A* CpG sites in cord blood monocytes, as determined in GSE212174 dataset [7]. Methylation in GSE212174 dataset was quantified using Infinium MethylationEPIC BeadChip (Illumina). Shown are methylation data (normalized β values) for CpG sites that overlapped with those analyzed in the PlaNS cohort: CpG sites **A.** #10 (cg03363743) and **B.** #14 (cg22584138) in *SLC6A4*, and **C.** -1665 (cg0207079) in *HTR2A*; methylation data for other CpG sites analyzed in the PlaNS cohort were not available. Each dot represents a single participant (N=29), with empty and filled dots indicating lean and obese mothers, respectively. Horizontal lines indicate median and interquartile range. $^{**}p<0.01$

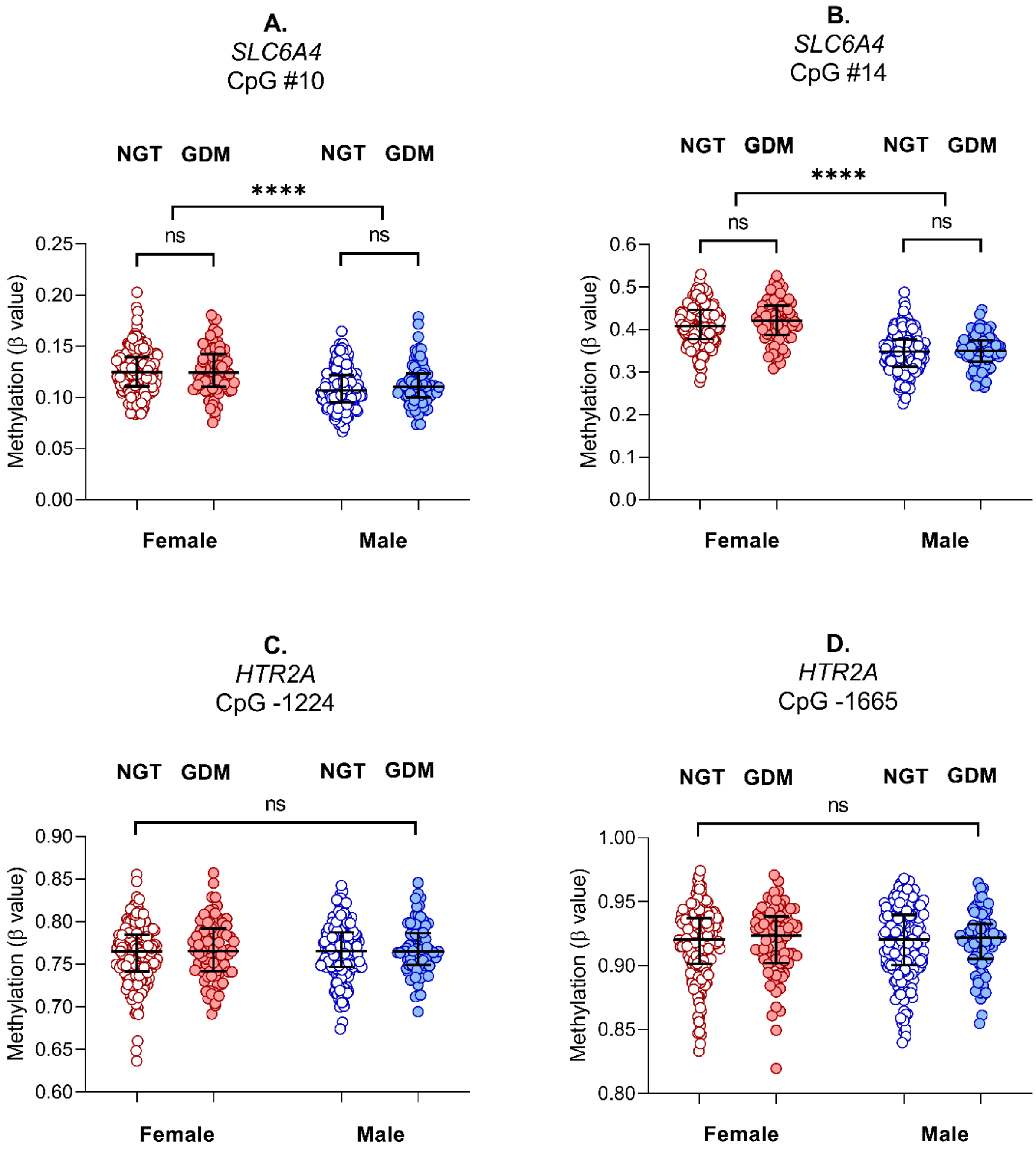

Figure S3. Association of newborn sex and maternal glucose tolerance status with methylation of *SLC6A4* and *HTR2A* CpG sites in cord blood cells, as determined in GSE141065 dataset [8]. Methylation in GSE141065 dataset was quantified using Infinium MethylationEPIC BeadChip (Illumina). Shown are methylation data (normalized β values) for CpG sites that overlapped with those analyzed in the PlaNS cohort: CpG sites **A.** #10 (cg03363743) and **B.** #14 (cg22584138) in *SLC6A4*, and CpG sites **C.** -1224 (cg27068143) and **D.** -1665 (cg0207079) in *HTR2A*; methylation data for other CpG sites analyzed in the PlaNS cohort were not available. Each dot represents a single participant (N=557), with empty and filled dots indicating mothers with normal glucose tolerance (NGT) and mothers with gestational diabetes mellitus (GDM), respectively. Horizontal lines indicate median and interquartile range. $^{****}p<0.0001$

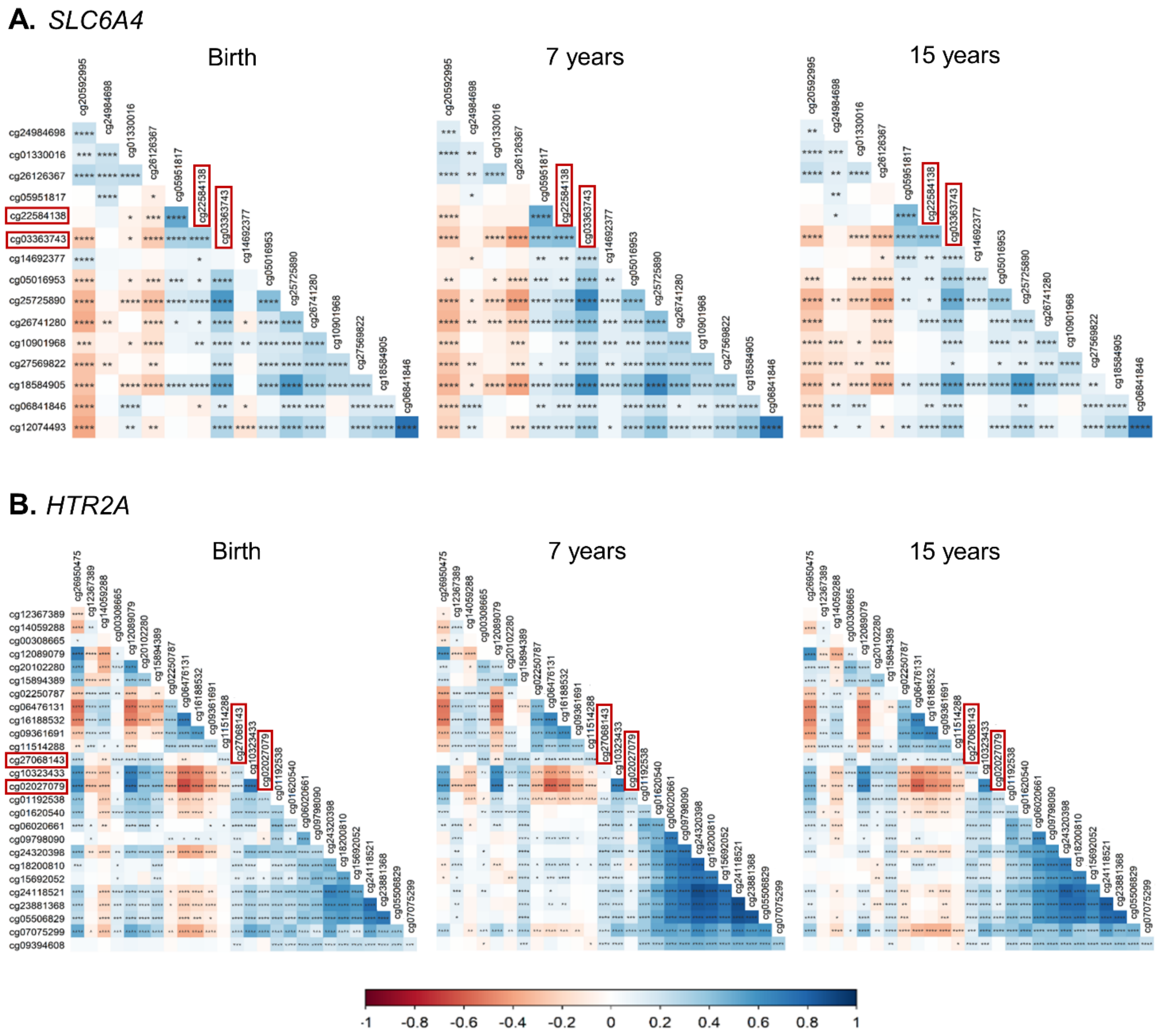

Figure S4. Correlation between methylation levels at CpG sites in **A.** *SLC6A4* and **B.** *HTR2A*, as observed in the ARIES cohort. Correlations for methylation levels in cord blood cells (left) and peripheral blood cells at ages 7 years (middle) and 15 years (right) are shown (N=808). The color of each square denotes the strength of correlation calculated by Spearman correlation coefficient, as indicated in the legend. Asterisks within each square indicate corresponding *p*-values: *$p<0.05$, **$p<0.01$, ***$p<0.001$, ****$p<0.0001$. CpG sites analyzed in the PlaNS cohort are indicated by red rectangles. The graph was created using the "corrplot" package of the R software [6].

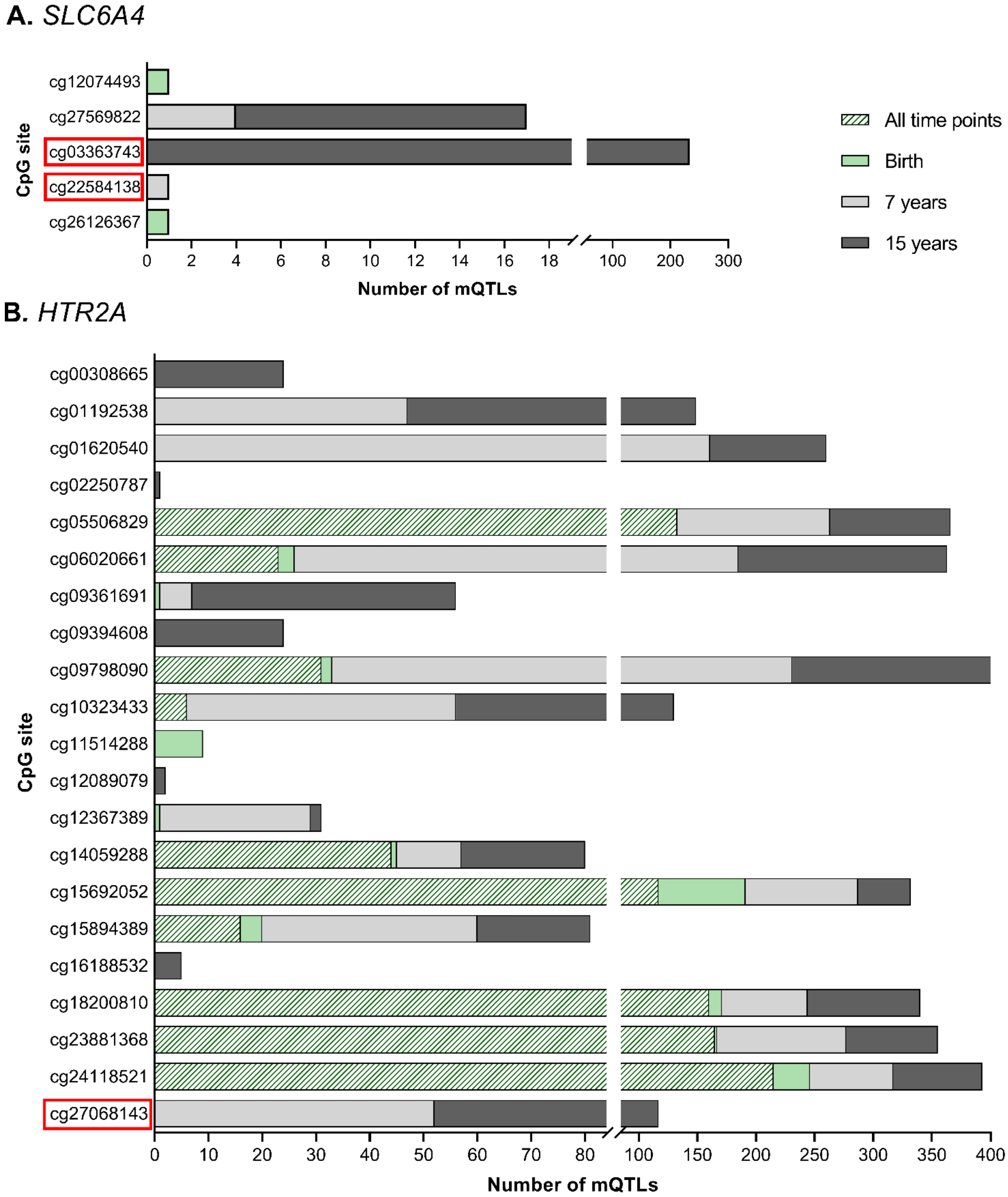

Figure S5. Temporal mapping of methylation quantitative trait loci (mQTLs) for Illumina 450K CpG sites in **A.** *SLC6A4* and **B.** *HTR2A* genes in the ARIES cohort. Annotations of CpG sites correspond to Illumina Infinium 450K identifiers (IDs). CpG sites studied in the PlaNS cohort are indicated by red rectangles. Only CpG sites associated with at least one mQTL are shown (5 of 16 for *SLC6A4* and 21 of 27 for *HTR2A*). The length of the bars represents the number of mQTLs associated with each of the CpG sites. Each bar is subdivided by the time points at which mQTLs were detected, as indicated in the legend. Data are from the mQTL database [9].

Supplementary references

1. Alfano R, Chadeau-Hyam M, Ghantous A, Keski-Rahkonen P, Chatzi L, Perez AE, et al. A multi-omic analysis of birthweight in newborn cord blood reveals new underlying mechanisms related to cholesterol metabolism. Metabolism. 2020;110:154292.

2. Philibert RA, Gunter TD, Beach SR, Brody G, Madan A. *MAOA* methylation is associated with nicotine and alcohol dependence in women. Am J Med Genet B Neuropsychiatr Genet. 2008;147B:565–70.

3. Shumay E, Logan J, Volkow ND, Fowler JS. Evidence that the methylation state of the monoamine oxidase A (*MAOA*) gene predicts brain activity of MAOA enzyme in healthy men. Epigenetics. 2012;7:1151–60.

4. Hranilovic D, Blazevic S, Stefulj J, Zill P. DNA methylation analysis of *HTR2A* regulatory region in leukocytes of autistic subjects. Autism Res. 2016;9:204–9.

5. Paquette AG, Lesseur C, Armstrong DA, Koestler DC, Appleton AA, Lester BM, et al. Placental *HTR2A* methylation is associated with infant neurobehavioral outcomes. Epigenetics. 2013;8:796–801.

6. Wei T, Simko V. R Package "Corrplot": Visualization of a Correlation Matrix (Version 0.84). 2017. Available from: https://github.com/taiyun/corrplot

7. Krause BJ, Vega-Tapia FA, Soto-Carrasco G, Lefever I, Letelier C, Saez CG, et al. Maternal obesity and high leptin levels prime pro-inflammatory pathways in human cord blood leukocytes. Placenta. 2023;142:75–84.

8. Antoun E, Kitaba NT, Titcombe P, Dalrymple KV, Garratt ES, Barton SJ, et al. Maternal dysglycaemia, changes in the infant's epigenome modified with a diet and physical activity intervention in pregnancy: Secondary analysis of a randomised control trial. PLoS Med. 2020;17:e1003229.

9. Gaunt TR, Shihab HA, Hemani G, Min JL, Woodward G, Lyttleton O, et al. Systematic identification of genetic influences on methylation across the human life course. Genome Biol. 2016;17:61.
